# Standardized tools for assessing balance and mobility in stroke clinical practice guidelines worldwide: A scoping review

**DOI:** 10.3389/fresc.2023.1084085

**Published:** 2023-02-21

**Authors:** Renato B. dos Santos, Annabelle Fiedler, Anchal Badwal, Jean Michelle Legasto-Mulvale, Kathryn M. Sibley, Olubukola A. Olaleye, Gudrun Diermayr, Nancy M. Salbach

**Affiliations:** ^1^ Master’s and Doctoral Programs in Physical Therapy, Universidade Cidade de Sao Paulo – UNICID, Sao Paulo, Brazil; ^2^ Rehabilitation Sciences Institute, University of Toronto, Toronto, ON, Canada; ^3^ School of Therapeutic Sciences, SRH University Heidelberg, Heidelberg, Germany; ^4^ Department of Physical Therapy, University of Toronto, Toronto, ON, Canada; ^5^ Department of Community Health Sciences, University of Manitoba, Winnipeg, MB, Canada; ^6^ George and Fay Yee Centre for Healthcare Innovation, University of Manitoba, Winnipeg, MB, Canada; ^7^ Department of Physiotherapy, Faculty of Clinical Sciences, College of Medicine, University of Ibadan, Ibadan, Nigeria; ^8^ The KITE Research Institute, University Health Network, Toronto, ON, Canada

**Keywords:** stroke rehabilitation, balance, mobility, clinical practice guideline, standardized assessment tools, implementation resources, country income

## Abstract

**Background:**

Use of standardized tools to assess balance and mobility limitations is a recommended practice in stroke rehabilitation. The extent to which clinical practice guidelines (CPGs) for stroke rehabilitation recommend specific tools and provide resources to support their implementation is unknown.

**Purpose:**

To identify and describe standardized, performance-based tools for assessing balance and/or mobility and describe postural control components challenged, the approach used to select tools, and resources provided for clinical implementation, in CPGs for stroke.

**Methods:**

A scoping review was conducted. We included CPGs with recommendations on the delivery of stroke rehabilitation to address balance and mobility limitations. We searched seven electronic databases and grey literature. Pairs of reviewers reviewed abstracts and full texts in duplicate. We abstracted data about CPGs, standardized assessment tools, the approach for tool selection, and resources. Experts identified postural control components challenged by each tool.

**Results:**

Of the 19 CPGs included in the review, 7 (37%) and 12 (63%) were from middle- and high-income countries, respectively. Ten CPGs (53%) recommended or suggested 27 unique tools. Across 10 CPGs, the most commonly cited tools were the Berg Balance Scale (BBS) (90%), 6-Minute Walk Test (6MWT) (80%), Timed Up and Go Test (80%) and 10-Meter Walk Test (70%). The tool most frequently cited in middle- and high-income countries was the BBS (3/3 CPGs), and 6MWT (7/7 CPGs), respectively. Across 27 tools, the three components of postural control most frequently challenged were underlying motor systems (100%), anticipatory postural control (96%), and dynamic stability (85%). Five CPGs provided information in varying detail on how tools were selected; only 1 CPG provided a level of recommendation. Seven CPGs provided resources to support clinical implementation; one CPG from a middle-income country included a resource available in a CPG from a high-income country.

**Conclusion:**

CPGs for stroke rehabilitation do not consistently provide recommendations for standardized tools to assess balance and mobility or resources to facilitate clinical application. Reporting of processes for tool selection and recommendation is inadequate. Review findings can be used to inform global efforts to develop and translate recommendations and resources for using standardized tools to assess balance and mobility post-stroke.

**Systematic Review Registration:**

https://osf.io/, identifier: 10.17605/OSF.IO/6RBDV.

## 1. Introduction

Stroke remains a major cause of disability globally (1). Approximately 38% of people with stroke in high-income countries, and up to 77% of people with stroke in low and middle-income countries, experience moderate or severe functional disability (2). Stroke-related impairments can result in low levels of physical activity (3), loss of independence (4–6), and falls (7). One of the most common problems after a stroke is balance and mobility limitations (8–10) which negatively impact performance of everyday activities (11). Balance can be defined as the ability to keep the center of mass within the base of support, and is a prerequisite to the maintenance of a sitting or standing posture, and mobility (12). Mobility is defined as changing body position, walking and moving (13). In fact, improving walking, a component of mobility, is one of the main rehabilitation goals among people with stroke and their caregivers (14–16). Hence, physical therapists (PTs) dedicate most of the time in a rehabilitation session on practicing mobility tasks compared with other activities (17, 18).

Assessing balance and mobility limitations using standardized assessment tools [i.e., tools with a specific testing protocol and scoring procedure (19)] is a critical aspect of high-quality and effective rehabilitation for individuals with stroke (20). Assessment tools used in clinical practice have three main purposes: to discriminate between individuals, to predict outcome or prognosis; and to monitor within-person change over time (21). Findings from assessment tools may also inform selection of treatment interventions, education of patients and families, and evaluations of readiness for discharge (22–25). Given the complexity of balance control, assessment tools have been developed to assist PTs with identifying the underlying postural control impairments that may account for poor balance and mobility (25, 26). Understanding the components of postural control challenged during the administration of individual assessment tools is expected to help align tool selection with the goals of therapeutic balance interventions (25).

The use of standardized assessment tools in physical therapy practice is inconsistent (27–32). Common barriers to the use of standardized assessment tools are lack of time, insufficient knowledge, lack of description of how to administer standardized assessment tools, and low perceived value of some instruments (24, 28, 29, 31–36). Additionally, the context in which PTs practice, such as the income level of a country, influences practice experiences (33). For example, a survey conducted in 2019 found that PTs practicing in Canada identified a lack of knowledge of which assessment tool to select and how to administer the assessment tool as primary barriers (33). In contrast, PTs practicing in India reported the unavailability of assessment tools and cost as key barriers (33). Facilitators to the use of standardized assessment tools for PTs practicing in Canada and India were known reliability and validity, familiarity with assessment instruments from PT training, and recommendations of assessment tools in clinical practice guidelines (CPGs) (33).

Recommendations for the use of assessment tools are inconsistent across CPGs (37). For example, results from a review of guidelines from low- and middle-income countries showed that assessment tools were not mentioned in three of six stroke guidelines (38). In a systematic review examining upper limb assessment recommendations in guidelines for people with neurological conditions (37), authors found that CPGs from Australia (39), UK (40), South Africa (41), Singapore (42), New Zealand (43) recommended using valid assessment tools without reference to specific tools to use. Moreover, recommendations to use specific assessment tools in CPGs from Estonia (44), the Netherlands (45), the UK (46), and the United States (47), respectively, do not align (37). In the last decade, work has been undertaken to establish recommended consensus-based core sets of assessment tools for research and clinical practice in rehabilitation post-stroke (20, 48, 49). It remains unknown, however, if these consensus-based core sets align with recommendations for assessment in CPGs worldwide. Frameworks for guideline development and implementation suggest that a guideline should clearly describe in detail the methods used for guideline development (50), such as the approach to selecting and recommending a specific assessment tool, and include resources to facilitate clinical implementation (51–54). Implementation resources could include administration protocols and guidance for interpretation of evaluation results in clinical practice. The extent to which CPGs for stroke rehabilitation recommend specific tools and provide resources to support their implementation is unknown.

To our knowledge, no previous studies have examined recommendations for use of standardized tools to assess balance and mobility, rationale for tool selection, and resources to support clinical application among existing stroke CPGs. This information could help to inform international efforts to develop a standardized set of CPG recommendations and resources to guide the assessment of balance and mobility post-stroke in low, middle, and high-income countries. Therefore, the objectives of this study were to: (1) identify standardized performance-based tools for the assessment of balance and mobility included in CPGs for stroke worldwide; (2) describe the postural control components challenged and instructions for using these tools; (3) describe the methods and criteria used to select and recommend these tools; (4) describe the resources that guideline developers provide to help clinicians implement these tools; and (5) present findings according to country income level.

## 2. Materials and methods

### 2.1. Protocol and registration

We conducted a scoping review following the five steps proposed by Arksey and O'Malley (55), and Levac (56) to develop the review protocol: (1) identifying the research question, (2) identifying relevant studies, (3) study selection, (4) charting the data, and (5) collating, summarizing, and reporting the results. We developed a protocol *a priori* and prospectively registered the protocol with the Open Science Framework (doi 10.17605/OSF.IO/6RBDV). We used the Preferred Reporting Items for Systematic Reviews and Meta-Analyses extension for Scoping Reviews (PRISMA-ScR) to guide reporting (57).

### 2.2. Eligibility criteria

We included documents meeting the following inclusion criteria: (1) document is a CPG; (2) recommendations target adults (age 18 years or older) with stroke [guidelines developed for a broader population (e.g., neurological) were included provided they specified people post-stroke as a sub-population]; (3) document includes recommendations on the delivery (e.g., assessment/treatment) of rehabilitation of balance and/or mobility; (4) document was published between January 2014 and December 2021 [the 7-year time frame was established based on recommended time intervals between guideline updates of between 2 and 5 years (58, 59) and considering the publication processing time]; and 5) document was written in English, French, German, Portuguese or Spanish as these were languages understood by review team members. Summaries or synopses of guidelines, or older versions of guidelines that had been updated, were excluded. See Supplementary File 1 for the operational definitions used in the review. We revised an eligibility criterion in the registered protocol related to the scope of the CPG to include CPGs with specific recommendations related to the assessment or treatment of balance and/or mobility. This decision was made due to CPGs focusing on rehabilitation of constructs (e.g., cognition) not relevant to the review, or CPGs that only mentioned the need for rehabilitation of balance and/or mobility without providing specific recommendations.

### 2.3. Information sources and search strategy

#### 2.3.1. Search of peer-reviewed literature

Using a validated search filter created by the Canadian Agency for Drugs and Technologies in Health (CADTH) (60, 61), and input from academic librarians, we developed and tailored a search strategy to seven scientific electronic databases: Medline, EMBASE, PEDro, Global Index Medicus, Cochrane Library, Guidelines International Network (GIN), and TRIP (Turning Research into Practice) Medical Database. The search strategies were translated using each electronic database's command language, controlled vocabulary, and appropriate search fields.

#### 2.3.2. Search of grey literature

To locate CPGs not indexed in the scientific electronic databases, we contacted member associations of World Physiotherapy (62) and the World Stroke Organization (63) to inquire about existing CPGs issued by their organization or country. An e-mail explaining the purpose of the study was sent to each association. Two reminder emails were sent 2 and 7 days later (64). In the case of no response, we manually searched each organization's website. We screened reference lists of included CPGs to identify additional CPGs. Supplementary File 2 presents the Ovid/Medline search strategy and the approach used to contact member associations of World Physiotherapy and the World Stroke Organization.

### 2.4. Selection of sources of evidence

We imported the identified records into EndNote X8 (Clarivate Analytics, Philadelphia, PA) and removed duplicates using Bramer et al.'s approach (65). To optimize consistency among reviewers (57), the six reviewers (RBS, AF, AB, OAO, GD, NMS) underwent a training process. The training consisted of reviewing the same subset of abstracts (*n* = 50) and full-text articles (*n* = 10), and then meeting to discuss the results and amend the screening form and guide before beginning the screening process. Given the high number of records retrieved, one reviewer screened the titles for potentially relevant records. The abstracts of a random sample of excluded titles (5%) were verified by the review team to ascertain the quality of the title screening process. Records that passed the title screening were imported into Covidence (66). Then, in pairs, the six reviewers independently reviewed all abstracts and full-text records. Disagreements regarding CPG eligibility were discussed with the review team, reasons for disagreement were explored, and final decisions on CPGs eligibility were made by consensus.

### 2.5. Data extraction and items

We developed a data extraction form and guide using Microsoft Excel. Two reviewers piloted the data extraction form and guide with 10 records and discussed the results to standardize the data extraction process. Subsequently, data extraction was conducted by one reviewer and verified by at least one other reviewer. We extracted data on: (1) characteristics of CPGs (e.g., title, authors, sponsoring organization, year of publication, country, language); (2) information about the assessment tools (e.g., name and/or version, measurement properties provided in the guideline, references listed for the tool, construct assessed, and timing of administration recommended); (3) methods and criteria used to select and recommend the tools (copied from CPGs verbatim); and (4) resources provided by guidelines to help end-users administering the tools.

### 2.6. Data synthesis and analysis

We classified a tool as assessing balance and/or mobility if the tool: (1) had a stated objective to assess balance and/or mobility outlined in the publication presenting its development and/or initial psychometric evaluation or is commonly used to assess balance and/or mobility as indicated by web-based knowledge syntheses (67, 68), and (2) scoring was based on the performance of a balance and/or mobility task. The primary focus of the measures identified was used to help classify a tool as one assessing balance, mobility, or balance and mobility. First, we identified and included assessment tools in the CPGs which had been included in a previous scoping review of measures of standing balance for adult populations conducted by Sibley et al. (69). Subsequently, the eligibility of the remaining tools was screened by two reviewers (GD, NMS) with expertise in the assessment of balance and mobility.

CPGs were classified as including or not including a standardized assessment tool. CPGs including standardized assessment tools were then classified as either “recommending” or “suggesting” the use of a balance and/or mobility assessment tool. Among these CPGs we then determined how frequently each tool was included, and the percentage of CPGs that described methods of selection and provided resources. For the 10mWT, we computed the frequency at which the tool was recommended based on the distance being timed (e.g., 5, 6 or 10 meters), as we considered these as distinct tools.

We identified the components of postural control challenged during the administration of each assessment tool using the following definitions of the nine components of postural control proposed by Sibley et al. (69) adapted from the Systems Framework for Postural Control (70): (1) static stability: ability to maintain position of the center of mass in unsupported stance when the base of the support does not change; (2) underlying motor systems (e.g., strength, coordination, postural alignment); (3) functional stability limits: ability to move the center of mass as far as possible in the anteroposterior or mediolateral directions within the base of support; (4) verticality: ability to orient appropriately with respect to gravity; (5) reactive postural control: ability to recover stability after an external perturbation to bring the center of mass within the base of support through corrective movements; (6) anticipatory postural control: ability to shift the center of mass before a discrete voluntary movement; (7) dynamic stability: ability to exert ongoing control of center of mass when the base of the support is changing; (8) sensory integration: ability to reweigh sensory information when input alters; and (9) cognitive influences: ability to maintain stability while responding to commands during the task or attend to additional tasks. For an assessment tool containing multiple subscales (i.e., Chedoke-McMaster Stroke Assessment Scale, Fugl-Meyer Assessment of Motor Recovery after Stroke, Rivermead Motor Assessment, Stroke Rehabilitation Assessment of Movement), we first identified the components of postural control challenged by each subscale designed to evaluate balance and/or mobility, and then determined the total number of unique components of postural control for the subscales combined. Tools were evaluated by one reviewer and verified by a second reviewer. We achieved consensus through discussion among reviewers with expertise in balance and mobility assessment and by reviewing the framework for postural control (70). Our evaluation of standing balance items was informed by identification of postural control components from a previous review (69). For the tools with one or more subscales, we report the frequency of recommendation and components of postural control as a single tool.

Additionally, when CPGs provided resources, we described the resources and additional instructions, the recommended time of administration, and level of recommendations reported. To examine the findings according to country income level, we classified CPGs as from a low-, middle- or a high-income country, according to income level definitions from the World Bank (71).

Upon completion of the data analysis, we emailed developers of CPGs that either recommended or suggested using specific standardized tools for assessing balance and/or mobility and invited them to verify the data, provided in a summary table, abstracted and synthesized from their guideline.

## 3. Results

### 3.1. Selection of sources of evidence

**Figure 1:**
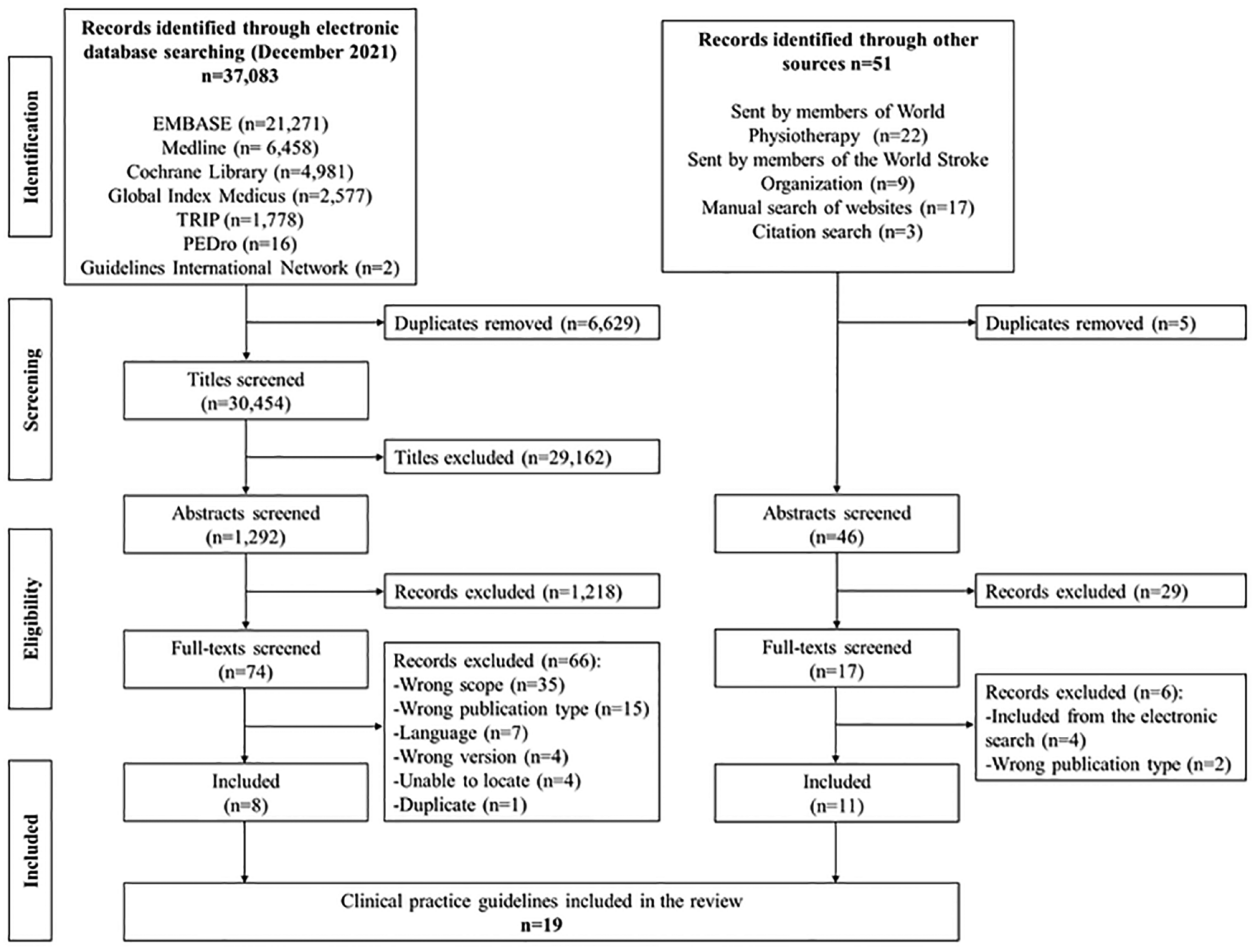
Results of guideline searching and selection.

The PRISMA-ScR flow diagram (57) in Figure 1 shows the results of the search and reasons for exclusion of full-text records. A total of 19 CPGs (20, 45, 47, 72–87) met the eligibility criteria and were included in our scoping review. Of the 19 included CPGs, 8 CPGs were located from bibliographic databases, and 11 CPGs from other sources (i.e., members of World Physiotherapy and the World Stroke Organization, and citation search of included CPGs).

### 3.2. Characteristics of sources of evidence

Table 1 presents the characteristics of the guidelines included in the review, the number of tools assessing balance and/or mobility specified, methods and criteria for selecting and recommending or suggesting assessment tools, and whether resources were provided. Of the 19 CPGs included, 12 (63%) (20, 45, 47, 72–74, 76, 78, 79, 82, 85, 87) were from high-income countries, and 7 (37%) (75, 77, 80, 81, 83, 84, 86) were from middle-income countries. CPGs were written in English (*n* = 14), German (*n* = 3), French (*n* = 1), and Spanish (*n* = 1). Publication dates ranged from 2014 to 2021, with 13 (68%) CPGs published during or after 2018. Ten (53%) CPGs (20, 45, 47, 72–74, 79, 80, 83, 86) either recommended (*n* = 6, 60%) (20, 45, 47, 72, 73, 83) or suggested (*n* = 4, 40%) (74, 79, 80, 86) using specific standardized tools for assessing balance and/or mobility. One (5%) CPG (75) recommended the use of standardized assessment tools without reference to specific tools; one (5%) CPG (81) included tools that did not assess balance and/or mobility; and seven (37%) CPGs (76–78, 82, 84, 85) did not include any recommendation for assessment. Of the 10 CPGs that included specific standardized tools for assessing balance and/or mobility, five (50%) (20, 45, 47, 72, 80) specified the approach for selecting and recommending the assessment tools, and 7 (70%) CPGs (20, 45, 47, 72–74, 86) provided resources designed to guide end-users with administering the assessment tools. Supplementary File 3 provides a list of all standardized assessment tools identified across CPGs.

**Table 1:** Characteristics of guidelines included in the review (*n* = 19).

Guideline name (Organization)	Country Income level Language Year of publication	No. of tools to assess balance and/or mobility identified in guideline	Description of how tools were selected and recommended	Resource(s) provided
*1. Accident vasculaire cérébral pertinence des parcours de rééducation/réadaptation après la phase initiale de l'AVC - Note de problématique (Haute Autorité de Santé)* (72)	France High-income French 2019	16 tools recommended	Selection based on two documents developed by the Ministry of Health and Social Services in Quebec (88), and Ministry of Health in France (89).	Yes
2. A core set of outcome measures for adults with neurologic conditions undergoing rehabilitation. (Academy of Neurologic Physical Therapy - American Physical Therapy Association) (20)	United States High-income English 2018	5 tools recommended	The development group consisted of 3 physical therapists with expertise in OMs. To determine the scope of the CPG, surveys were conducted to assess the needs and priorities of consumers (e.g., people with stroke, spinal cord injury, multiple sclerosis, and Parkinson disease) and physical therapist members of the ANPT. OMs were identified through recommendations of the ANPT-EDGE task forces (rating from 2 to 4/4 where “4” indicated that the measure had excellent psychometric properties and clinical utility in the target condition). A systematic review of the literature on the OMs was conducted and additional OMs were identified; the literature search was repeated on these measures. Two trained reviewers appraised the articles using a modified version of the COSMIN checklist. Methodological quality and the strength of statistical results were determined. To be included, measures had to: (a) be generic/not condition-specific, (b) have >75% of the items or questions aiming to assess balance, gait, transfers, and patient-stated goals, (c) be used in 2 or more neurologic populations, (d) evaluate change, (e) with excellent clinical utility (had to be free, require equipment commonly available in a clinic, and take ≤20 min to administer), (f) reliability and data available in at least one article to support the assessment of change (e.g., minimal detectable change and minimal clinically important difference), (g) level I evidence of excellent internal consistency and/or reliability and standard error of measurement/minimal detectable change data in 2 or more populations and 3 condition categories (acute, chronic stable, and chronic progressive). When more than one measure in a construct area had substantial supporting evidence, the measure with the strongest psychometric properties across diagnostic groups was selected.	Yes
3. Canadian stroke best practice recommendations: Rehabilitation, recovery, and community participation following stroke. Part one: Rehabilitation and recovery following stroke; 6th edition update 2019. (Heart and Stroke Foundation) (74)	Canada High-income English and French* 2020	9 tools suggested	The tools were selected based on current available evidence of psychometric properties, validity and reliability within the stroke context, accessibility and confirmed by consensus of the expert writing group^†^	Yes
4. Chinese Stroke Association guidelines for clinical management of cerebrovascular disorders: executive summary and 2019 update of clinical management of stroke rehabilitation. (Chinese Stroke Association Stroke Council) (75)	China Middle-income English 2020	0	Not applicable	Not applicable
5. Clinical guidelines for stroke management. (Stroke Foundation) (76)	Australia High-income English Living CPG^‡^	0	Not applicable	Not applicable
6. Clinical practice guideline for the diagnosis, treatment and rehabilitation of the acute ischemic stroke episode in older than 18 years: from recommendations to its implementation. (Colombian General Social Security System for Health) (77)	Colombia Middle-income English 2015	0	Not applicable	Not applicable
7. Clinical practice guideline to improve locomotor function following chronic stroke, incomplete spinal cord injury, and brain injury. (American Physical Therapy Association) (78)	United States High-income English 2020	0	Not applicable	Not applicable
8. *DEGAM Leitlinie-S3: Schlaganfall. (Deutsche Gesellschaft für Allgemeinmedizin und Familienmedizin e.V*.) (79)	Germany High-income German 2020	2 tools suggested	Not reported	No
9. *Guía de práctica clínica fisioterapéutica para la evaluación y tratamiento de pacientes con enfermedades cerebrovasculares en los primeros seis meses de la enfermedad. (Asociación Colombiana de Fisioterapia; and Asociación Colombiana de Facultades de Fisioterapia*) (80)	Colombia Middle-income Spanish 2021	6 tools suggested	Team of physical therapists with expertise in neurorehabilitation and critical appraisal and epidemiologists participated in the search, review, evaluation and synthesis of scientific literature from 2016 onward, including existing guidelines. Three focus groups including patients and caregivers were held to validate the scope of the guideline and prioritize outcomes. Experts and caregivers used GRADE criteria and a 9-point ordinal scale to classify outcomes as critical (7-9 points), key for decision-making; important (4-6 points), not key for decision-making; not important (1-3 points) not recommended. Priority outcomes were balance, upper limb function, lower limb function (including gait). A literature search guided by the PICO question “What are the tests or measurement instruments with the best psychometric properties to assess motor function in adults older than 18 years after a stroke in the first six months?” was conducted. Given the diversity of assessment tools and insufficient evidence of sensitivity, specificity, validity and reliability, the team decided to analyze tools used to assess the effectiveness of the interventions described in articles appraised for treatment recommendations. Tools were described as having good psychometric properties and available in Spanish.	No
10. Guidelines for adult stroke rehabilitation and recovery: A guideline for healthcare professionals from the American Heart Association/American Stroke Association. (American Heart Association/American Stroke Association) (47)	United States High-income English 2016	6 tools recommended	Measures reviewed in the Evidence-Based Review of Stroke Rehabilitation report as of November 2012.	Yes
11. Guidelines for prevention and management of stroke. (National programme for prevention and control of cancer, diabetes, cardiovascular diseases & stroke) (81)	India Middle-income English 2019	0	Not applicable	Not applicable
12. KNGF Guideline Stroke. (*Koninklijk Nederlands Genootschap voor Fysiotherapie)* (45)	The Netherlands High income English and Dutch* 2014	6 tools recommended	Selected on the basis of reliability, responsiveness, predictive and construct validity, and their practical feasibility.	Yes
13. National clinical guideline for stroke: fifth edition. (Intercollegiate Stroke Working Party, Royal College of Physicians of London) (40)	United Kingdom High-income English 2016	0	Not applicable	Not applicable
14. Philippine Academy of Rehabilitation Medicine (PARM): Clinical practice guideline on stroke rehabilitation (Updated: 2017). (83)	Philippines Middle-income English 2019	2 tools recommended	Not reported	No
15. Recommendations for the early management of acute ischemic stroke: A consensus statement for healthcare professionals from the Indian Stroke Association. (Indian Stroke Association) (84)	India Middle-income English 2018	0	Not applicable	Not applicable
16. *Rehabilitation der Mobilität nach Schlaganfall (ReMoS) - S2e-Leitlinie. (*ReMoS working group) (85)	Germany High-income German 2015	0	Not applicable	Not applicable
17. *Rehabilitation von sensomotorischen Störungen.* (*Deutsche Gesellschaft für Neurologie*) (73)	Germany High-income German 2018	10 tools recommended	Not reported	Yes
18. South African-contextualised stroke rehabilitation guideline (SA-CSRG). (National Department of Health and Stellenbosch University) (86)	South Africa Middle-income English 2019	6 tools suggested	Not reported	Yes
19. VA/DoD clinical practice guideline for the management of stroke rehabilitation (version 4.0). (Department of Veterans Affairs and the Department of Defense) (87)	United States High-income English 2019	0	Not applicable	No

No.: number; OM: outcome measure; ANPT: Academy of Neurologic Physical Therapy; EDGE: Evidence Database to Guide Effectiveness; COSMIN: COnsensus-based Standards for the selection of health Measurement INstruments.

* We extracted data from the English version.

^†^ Information provided by the CPG developer during data accuracy check.

^‡^ Guideline was first published in 2017 and recommendations are continually reviewed and updated in response to new evidence.

### 3.3. Synthesis of results

Five (50%) of the 10 CPG developers that either recommended or suggested using specific standardized tools for assessing balance and/or mobility responded to our request to review. All five CPG developers confirmed that the information was accurate, and two suggested minor clarifications related to the approach used to select and recommend the assessment tools.

### 3.4. Standardized tools for assessing balance and/or mobility included in stroke CPGs

Table 2 presents the names of the balance/mobility tools specified, timing and additional instructions for administration, and the level of recommendation. Across 10 CPGs that specified assessment tools, we identified 27 unique tools for assessing balance (*n* = 13), mobility (*n* = 13), or balance and mobility (*n* = 1). The number of balance and/or mobility tools included in each CPG varied from 2 (79, 83) to 16 (72). Across 10 CPGs, the assessment tools most commonly specified were the Berg Balance Scale (BBS) (90%) (20, 45, 47, 72–74, 80, 83, 86), the 6-Minute Walk Test (6MWT) (80%) (20, 45, 47, 72–74, 79, 86), the Timed Up and Go Test (TUG) (80%) (45, 47, 72–74, 79, 80, 86), and the 10-Meter Walk Test (10mWT) (70%) (45, 47, 72–74, 83, 86). Conversely, fifteen tools (56%) were only recommended once across the 10 CPGs.

**Table 2:** Balance and mobility assessment tools, resources, instructions, and level of recommendation included in CPGs (*n* = 10).

Guideline	Tools to assess balance and/or mobility	Timing of administration recommended	Level of recommendation *Additional instructions*	Resources description
*Accident vasculaire cérébral Pertinence des parcours de rééducation/réadaptation après la phase initiale de l’AVC - Note de Problématique* (72)	BBS CMSA EPA EPD MAS Mini-BESTest PASS POMA RMA SMES Step Test STREAM TCT TUG 6MWT 10mWT	• Initial assessment within 24 to 48 h • Reassessment after acute phase to monitor change	Not reported *The evaluation should be according to the International Classification of Functioning, Disability and Health domains, as well as considering the elements of context (social, family, professional, cultural environment).*	Provides references to external free online documents containing a description of the tool, purpose of the tool, timing/duration of administration, administrator qualifications, data collection form, and references.
A core set of outcome measures for adults with neurologic conditions undergoing rehabilitation (20)	BBS FGA 5TSTS 6MWT 10mWT^†^	• At admission • At discharge • When possible, between these periods	For BBS:* Acute, chronic stable, and chronic progressive conditions: Evidence quality: I Recommendation strength: strong For FGA:* Acute, chronic stable conditions: Evidence quality: I Recommendation strength: strong Chronic progressive conditions: Evidence quality: I Recommendation strength: moderate For 5TSTS:* Evidence quality: V Recommendation strength: best practice For 6MWT:* Acute conditions: Evidence quality: V Recommendation strength: best practice Chronic stable conditions: Evidence quality: I Recommendation strength: moderate Chronic progressive conditions: Evidence quality: I Recommendation strength: strong **For 10mWT**^†^:* Acute conditions: Evidence quality: V Recommendation strength: best practice Chronic stable, chronic progressive conditions Evidence quality: I Recommendation strength: strong *1. To administer the assessment tools with patients who have goals and the capacity to improve transfers, balance, and/or gait, to assess changes over time; for patients who are unable to complete one or more core set OMs (e.g., a patient unable to walk who cannot complete the gait OMs) a score of 0 should be documented*. *2. To discuss the purpose of the assessments, their results, and how these results influence treatment options*. *3. To use the recommended assessment tools to assess change over time in a patient's balance, gait and transfers.*	Provides information under the following subheadings: Action statement (outcome assessed, indications for/timing of assessment); Aggregate evidence quality and strength; benefits (e.g., administration time, equipment); risk, harm, and cost; benefit-harm assessment; value judgments; intentional vagueness; role of patient preferences; exclusions; quality improvement; implementation and audit; supporting evidence and clinical interpretation (comprehensive summary of evidence of psychometric properties and interpretation); research recommendations. Provided hyperlinks to external free online documents containing an overview of the tool, number of items, scoring, equipment, duration of administration, cost, instructions for administration, what constitutes change, clinical interpretation of score, additional recommendations, common questions and variations, physical therapy report card, supporting references.
Canadian stroke best practice recommendations: Rehabilitation, recovery, and community participation following stroke. Part one: Rehabilitation and recovery following stroke; 6th edition update 2019. (74)	BBS COVS FRT Mini-BESTest RMA STREAM TUG 6MWT 10mWT	Not reported	Not applicable *The 5-meter or 10-meter gait speed test may be used as the most basic measurement for those not able yet to do 6-minute walk test.*	Provides a table describing the source reference, purpose, items and administration, additional considerations (e.g., administration requirements, clinical interpretation, evaluator training), availability (i.e., if available for free or purchase with hyperlinks to websites and mobile applications).
*DEGAM Leitlinie-S3: Schlaganfall.* (79)	TUG 6MWT	Not reported	Not applicable *Not reported*	Not reported
*Guía de práctica clínica fisioterapéutica para la evaluación y tratamiento de pacientes con enfermedades cerebrovasculares en los primeros seis meses de la enfermedad* (80)	BBS FMA Balance test FRT POMA RMA TUG	For patients ≤6 months post-stroke: • At the beginning of treatment • At end of treatment	Not applicable *Valid and reliable physiotherapeutic evaluation tests and measures are presented for the objective assessment of physical qualities and functional progression of patients with cerebrovascular disease*. *It is important to consider the cognitive and sensory capacity of patients (visual, auditory, vestibular) as these can change the performance of stability tasks.*	Not reported
Guidelines for adult stroke rehabilitation and recovery: A guideline for healthcare professionals from the American Heart Association/American Stroke Association (47)	BBS FRT TUG 6MWT 10mWT or 5mWT	Not reported	Not reported *To select a single tool for each construct; as it is often unnecessary to use >1 tool.*	Provides a table describing the construct, name of measure, comments (brief description of measure and, in some cases, clinical interpretation), time to administer, references for further information.
KNGF Guideline Stroke (45)	BBS TCT TIS TUG 6MWT 10mWT	In the first 6 months, the BBS, TCT, 10mWT are administered: • On initial evaluation • At end of first week • After 3 months • After 6 months • At end of treatment	Not reported *1. To select 1 or more tools based on the patient's physical condition, the severity of the stroke, and treatment goals, and/or use them based on own clinical reasoning;* *2. Not all the recommended assessment tools have to be taken during the same session;* *3. To use screening tools to detect and report impairments of body functions or environmental factors that do not primarily belong to PTs’ domain but may affect the physical therapy treatment*. *4. During acute phase (<6 months), a prognosis for walking ability can be established based on sitting balance (a score of 25 points on the sitting balance item of the TCT) and mild paresis of the leg (Motricity Index ≥25 points or a score of ≥19 on the motor part of the Fugl-Meyer Assessment for the lower extremity);* *5. During the chronic phase (>6 months) an indication of possible further changes in walking ability for patients who have a Functional Ambulation Categories score of 3+ at 6 months after the stroke can be obtained by the 10mWT at comfortable speed every 6 months. A meaningful change can be defined as a change in the walking speed of at least 0.16 m/s relative to the speed attained 6 months after the stroke.*	Provides tables describing the tools according to the International Classification of Functioning, Disability and Health domains, the construct, timing of administration, determinants and points for establishing a functional prognosis for walking ability, dexterity and basic activities of daily living. Provides a hyperlink to webpage (written in Dutch) with a standardized description of the tool including an overview, authors, explanation form (purpose, availability, psychometric evidence, feasibility, clinical interpretation, other information, references), measuring instrument (instructions for administration), instruction manual, other (e.g., calculation tool), target audience, type of measurement instrument, functions, body regions, diseases.
Philippine Academy of Rehabilitation Medicine (PARM): Clinical practice guideline on stroke rehabilitation (Updated: 2017) (83)	BBS 10mWT	Not reported	Not reported *At a minimum, 3 tools should be used (e.g., Functional Independence Measure mobility items, BBS, and the 10mWT) to assess gait velocity, functional ambulation classification, and assistance needed during daily activities.*	No
*Rehabilitation von sensomotorischen Störungen* (73)	BBS BBT DGI FRT MAS MCA TUG TCT 6MWT 10mWT	Not reported	Not reported *The diagnostic process should be guided by the International Classification of Functioning, Disability and Health.*	Provides references to two books to obtain detailed descriptions of the tools.
South African-contextualised stroke rehabilitation guideline (SA-CSRG) (86)	BBS FRT TUG 6MWT 10mWT or 5mWT	Not reported	Not applicable Not reported	Provides a table describing the name of the tool, minimal important difference, evidence base (references), other considerations (details of referenced studies). Provides a table taken from the AHA/ASA guideline describing the construct, name of measure, comments (brief description of measure and, in some cases, clinical interpretation), time to administer, references for further information.

BBS: Berg Balance Scale; BBT: Bohannon Balance Test; CMSA: Chedoke-McMaster Stroke Assessment; COVS: Clinical Outcome Variables Scale; DGI: Dynamic Gait Index; EPA: *l’indice d’équilibre postural assis*; EPD: *l’indice d’équilibre postural debout;* FGA: Functional Gait Assessment; FMA: Fugl-Meyer Assessment; FRT: Functional Reach Test; MAS: Motor Assessment Scale; MCA: Motor Club Assessment; Mini-BESTest: Mini Balance Evaluation Systems Test; PASS: Postural Assessment Scale for Stroke Patients; POMA: Performance Oriented Mobility Assessment; RMA: Rivermead Motor Assessment; SMES: Sødring Motor Evaluation of Stroke Patients; STREAM: Stroke Rehabilitation Assessment of Movement; TCT: Trunk Control Test; TIS: Trunk Impairment Scale; TUG: Timed Up and Go Test; 5mWT: 5-Meter Walk Test; 5TST: 5-Times Sit-to-Stand Test; 6MWT: 6-Minute Walk Test; 10mWT: 10-Meter Walk Test.

* Evidence quality I: evidence obtained from at least one high-quality (>50% critical appraisal score) study of psychometric properties; Evidence quality V: expert opinion (or best practice). Recommendation strength - Strong: a preponderance of level I studies, but at least 1 level I study directly on the topic supports the recommendation; Recommendation strength - Moderate: A preponderance of level II studies, but at least 1 level II study directly on the topic supports the recommendation; Recommendation strength – Best practice: best practice based on expert opinion (review papers, white papers, consensus documents) developed by various methodologies (e.g., Delphi and RAND) and the clinical experience of the guideline development group.

^†^ CPG recommends the 10-Meter Walk Test; however, the protocol for administration indicates that the time to walk the middle 6-meter section of the 10-meter walkway is documented and used to calculate walking speed.

Of the six CPGs (20, 45, 47, 72, 73, 83) in which assessment tools were recommended, only one (20) reported the level of recommendation. The level of recommendation included the level of evidence (I-V), and strength of the recommendation (weak, moderate, or strong) for three subgroups of patients (acute, chronic stable, and chronic progressive neurological conditions).

Table 3 describes the components of postural control challenged by the activities required to perform the assessment tools. Of the 27 unique tools, 13 tools (48%) challenge between four and six components of postural control, 11 (41%) challenge two or three components, two tools (7%) challenge seven components, and one tool (4%) challenges eight components of postural control. The three most frequently challenged components were: underlying motor systems (27 tools, 100%); anticipatory postural control (26 tools, 96%); and dynamic stability (23 tools, 85%). The three components least frequently challenged were reactive postural control (4 tools, 15%); verticality (3 tools, 11%); and cognitive influence (3 tools, 11%).

**Table 3:** Primary focus and components of postural control challenged for standardized tools for assessing balance and mobility included in stroke clinical practice guidelines.

Assessment tool or subscale	Primary focus	Components of postural control challenged
1. Berg Balance Scale*	Balance	• Static stability • Underlying motor systems • Functional stability limits • Anticipatory postural control • Dynamic stability • Sensory integration
2. Bohannon Balance Test	Balance	• Static stability • Underlying motor system
3.1 Chedoke-McMaster Stroke Assessment Scale: Postural control (Impairment inventory)	Balance	• Static stability • Underlying motor systems • Functional stability limits • Verticality • Anticipatory postural control • Dynamic stability
3.2 Chedoke-McMaster Stroke Assessment Scale: Activity inventory	Balance	• Static stability • Underlying motor systems • Anticipatory postural control • Dynamic stability • Sensory integration
4. Clinical Outcome Variables Scale	Mobility	• Static stability • Underlying motor systems • Anticipatory postural control • Dynamic stability
5. Dynamic Gait Index*	Mobility	• Underlying motor systems • Anticipatory postural control • Dynamic stability • Sensory integration • Cognitive influences
6. Fugl-Meyer Assessment of Motor Recovery after Stroke: Balance subscale	Balance	• Static stability • Underlying motor systems • Reactive postural control • Anticipatory postural control
7. Functional Gait Assessment*	Mobility	• Underlying motor systems • Anticipatory postural control • Dynamic stability • Sensory integration • Cognitive influences
8. Functional Reach Test*	Balance	• Underlying motor systems • Functional stability limits • Anticipatory postural control
9. *l’indice d’équilibre postural assis* (sitting postural balance index)	Balance	• Static stability • Underlying motor systems • Reactive postural control • Anticipatory postural control • Dynamic stability
10. *l’indice d’équilibre postural debout* (standing postural balance index)	Balance	• Static stability • Underlying motor systems • Anticipatory postural control
11. Mini-BESTest*	Balance	• Static stability • Underlying motor systems • Verticality • Reactive postural control • Anticipatory postural control • Dynamic stability • Sensory integration • Cognitive influences
12. Motor Assessment Scale	Mobility	• Static stability • Underlying motor systems • Anticipatory postural control • Dynamic stability
13. Motor Club Assessment: Functional movement activities	Mobility	• Static stability • Underlying motor systems • Anticipatory postural control • Dynamic stability
14. Performance Oriented Mobility Assessment*	Balance and Mobility	• Static stability • Underlying motor systems • Functional stability limits • Reactive postural control • Anticipatory postural control • Dynamic stability • Sensory integration
15. Postural Assessment Scale for Stroke Patients*	Balance	• Static stability • Underlying motor systems • Anticipatory postural control • Dynamic stability
16.1 Rivermead Motor Assessment: Section A - Gross function	Mobility	• Static stability • Underlying motor systems • Anticipatory postural control • Dynamic stability
16.2 Rivermead Motor Assessment: Section B - Leg and trunk section	Mobility	• Underlying motor systems • Anticipatory postural control • Dynamic stability
17. Sødring Motor Evaluation of Stroke Patients	Mobility	• Underlying motor systems • Functional stability limit • Anticipatory postural control • Dynamic stability
18. Step Test*	Balance	• Underlying motor systems • Anticipatory postural control • Dynamic stability
19. Stroke Rehabilitation Assessment of Movement: Mobility section	Mobility	• Static stability • Underlying motor systems • Anticipatory postural control • Dynamic stability
20. Timed Up and Go Test*	Mobility	• Underlying motor systems • Anticipatory postural control • Dynamic stability
21. Trunk Control Test	Balance	• Underlying motor systems • Anticipatory postural control • Dynamic stability
22. Trunk Impairment Scale	Balance	• Static stability • Underlying motor systems • Functional stability limits • Verticality • Anticipatory postural control • Dynamic stability
23. 5-Times Sit-to-Stand Test*	Mobility	• Underlying motor systems • Anticipatory postural control • Dynamic stability
24. 6-Minute Walk Test	Mobility	• Underlying motor systems • Anticipatory postural control • Dynamic stability
25. 10-Meter Walk Test: 10-meter distance timed (45, 47, 72–74, 83, 86)	Mobility	• Underlying motor systems • Anticipatory postural control • Dynamic stability
26. 5-Meter Walk Test: 5-meter distance timed (47, 86)		
27. 6-Meter Walk Test: 6-meter distance timed (20)^†^		

* Included in scoping review of standing balance measures by Sibley et al. (69).

^†^ CPG recommends the 10-Meter Walk Test; however, the protocol for administration indicates that the time to walk the middle 6-meter section of the 10-meter walkway is documented and used to calculate walking speed.

Four CPGs specified the timing of assessment. Two CPGs (72, 80) indicated to assess at two timepoints (within 24–48 h post-stroke and reassess after the acute phase to monitor change or at start and end of treatment). One CPG (20) specified three timepoints (on admission, at discharge, and in-between if possible). One CPG (45) specified five timepoints within the first six months post-stroke (initial evaluation, end of first week, after 3 months, after 6 months, end of treatment).

Table 2 presents additional instructions provided in 8 CPGs. Six (60%) CPGs provided instructions to end-users to select appropriate assessment tools (20, 45, 47, 72, 73, 83). Two CPGs (73) (72) recommended that the assessment should be guided by the International Classification of Functioning (ICF). One CPG (47) recommended selecting a single tool for each construct, one CPG (45) suggests PTs to select one or more of the recommended assessment tools, and one CPG (83), recommended that at a minimum, three assessment tools should be used. Finally, one CPG (20) recommended clinicians to administer six assessment tools in a core set to patients who have goals and the capacity to improve transfers, balance, and/or gait (Table 2).

### 3.5. Methods and criteria used to select and recommend the assessment tools

Table 1 presents the information provided in each CPG on describing how tools were selected and/or recommended or suggested. Across the 5 CPGs (20, 45, 47, 72, 80) that provided information, CPG developers most commonly identified psychometric properties as a basis for tool selection as noted in four CPGs (20, 47, 72, 80). Additional or alternative criteria for recommending tools included clinical utility/practical feasibility (20, 45) (e.g., free, requires equipment commonly available, takes ≤20 min to administer), interpretability (20), use in research to evaluate recommended treatments (80), documents developed by government health ministries (72), and availability of a translated version of the selected tools (80).

### 3.6. Resources provided by guidelines

Table 2 describes the resources to help end-users administer the assessment tools provided by seven (70%) CPGs (20, 45, 47, 72–74, 86). Two CPGs (47, 86) included a table with resources, two CPGs (45, 72) provided a link to external online resources, and two CPGs (20, 74) included resources in the guideline and provided a link to external online resources. One CPG (73) provides references to two books to obtain detailed descriptions of the tools. In general, the resources provided instructions on how to administer the assessment tools (e.g., number of items, time to complete, equipment, logistics), supporting evidence, and clinical interpretation for the assessment tools (e.g., cut-off scores and normative values). See the Supplementary File 2 for a table describing resources provided by each guideline in detail.

### 3.7. Characteristics of guidelines by country income level

Table 4 describes the characteristics of CPGs by country income level. Of the 10 CPGs including balance and/or mobility assessment tools, seven (70%) (20, 45, 47, 72–74, 79) were developed in high-income countries and three (30%) (80, 83, 86) in middle-income countries. No CPG were developed in low-income countries. Of the 27 assessment tools identified across countries, eight (30%) were specified in CPGs from both middle- and high-income countries. These tools were the BBS, Functional Reach Test, Performance Oriented Mobility Assessment, TUG, Rivermead Motor Assessment, 5- or 10-Meter Walk Test, and the 6MWT. In middle- and high-income countries, the top tool cited was the BBS (3/3 CPGs), and 6MWT (7/7 CPGs), respectively. Only one (80) of the five CPGs that described the approach for selecting and recommending the assessment tools was from a middle-income country, which included mention of the availability of assessment tools in Spanish. Lastly, of the seven CPGs providing resources to guide end-users in administering the assessment tools, only one (86) was from a middle-income country.

**Table 4:** Characteristics of guidelines that include balance and/or mobility assessment tools analyzed by country income level.

Characteristic	CPGs from middle-income countries (*n* = 3)	CPGs from high-income countries (*n* = 7)	All CPGs (*n* = 10)
	*N* (%)		
Language			
English	2 (67)	4 (57)	6 (60)
French	0	1 (14)	1 (10)
German	0	2 (29)	2 (20)
Spanish	1 (33)	0	1 (10)
CPGs describe the approach for selecting and recommending the assessment tools	1 (33)	4 (57)	5 (50)
CPGs provide resources for administration and/or interpretation	1 (33)	6 (86)	7 (70)
Standardized tools for assessing balance and mobility included in the CPGs			
1. Berg Balance Scale	3 (100)	6 (86)	9 (90)
2. Timed Up and Go Test	2 (67)	6 (86)	8 (80)
3. 6-Minute Walk Test	1 (33)	7 (100)	8 (80)
4. 10-Meter Walk Test	2 (67)	5 (71)	7 (70)
5. Functional Reach Test	2 (67)	3 (43)	5 (50)
6. Rivermead Motor Assessment: Section A - Gross function; Section B - Leg and trunk section	1 (33)	2 (29)	3 (30)
7. Trunk Control Test	0	3 (43)	3 (30)
8. Mini-BESTest	0	2 (29)	2 (20)
9. Motor Assessment Scale	0	2 (29)	2 (20)
10. Performance Oriented Mobility Assessment	1 (33)	1 (14)	2 (20)
11. Stroke Rehabilitation Assessment of Movement: Mobility section	0	2 (29)	2 (20)
12. 5-Meter Walk Test	1 (33)	1 (14)	2 (20)
13. Bohannon Balance Test	0	1 (14)	1 (10)
14. Chedoke-McMaster Stroke Assessment Scale: Postural control (Impairment inventory); Activity inventory	0	1 (14)	1 (10)
15. Clinical Outcome Variables Scale	0	1 (14)	1 (10)
16. Dynamic Gait Index	0	1 (14)	1 (10)
17. Fugl-Meyer Assessment of Motor Recovery after Stroke: Balance subscale	1 (33)	0	1 (10)
18. Functional Gait Assessment	0	1 (14)	1 (10)
19. *l’indice d’équilibre postural assis*	0	1 (14)	1 (10)
20. *l’indice d’équilibre postural debout*	0	1 (14)	1 (10)
21. Motor Club Assessment: Functional movement activities	0	1 (14)	1 (10)
22. Postural Assessment Scale for Stroke Patients	0	1 (14)	1 (10)
23. Sødring Motor Evaluation of Stroke Patients	0	1 (14)	1 (10)
24. Step Test	0	1 (14)	1 (10)
25. Trunk Impairment Scale	0	1 (14)	1 (10)
26. 5-Times Sit-to-Stand Test	0	1 (14)	1 (10)
27. 6-Meter Walk Test*	0	1 (14)	1 (10)

CPG: clinical practice guideline.

* CPG recommends the 10-Meter Walk Test; however, the protocol for administration indicates that the time to walk the middle 6-meter section of the 10-meter walkway is documented and used to calculate walking speed.

## 4. Discussion

Approximately half of CPGs from middle- and high-income countries with recommendations on the rehabilitation of balance and mobility post-stroke recommend or suggest a standardized tool for assessing balance and/or mobility. Although a large number (i.e., 27) of tools are identified across CPGs, the BBS, 6MWT, TUG, and 10mWT are most commonly listed. Despite the variability in tools, the activities required in the tools overlap in terms of the components of postural control they challenge, with a high proportion of tools challenging underlying motor systems, anticipatory postural control, and dynamic stability. Only half of CPGs specifying tools provide information on how tools were selected. Selection approaches vary widely and detailed descriptions are lacking. Providing a level of recommendation for assessment tools included in CPGs is rare. Description of an overall approach to clinical assessment is inconsistent. There is a gap in resources shared to facilitate the use of standardized assessment tools, especially in CPGs from middle-income countries.

The wide range of tools included in the CPGs reflects the plethora of existing tools to assess balance and mobility. Previous systematic reviews have identified multiple measures of sitting balance used for people after stroke (90), with over 60 different measures of standing balance in the adult population (69), and over 30 measures of mobility for older adults (91). We found that the assessment tools included in at least 70% of CPGs (i.e., BBS, 6MWT, TUG, and 10mWT) are consistent with the tools most frequently used in clinical practice as indicated by clinician surveys conducted in Canada (92–94), Colombia (95), Ghana (27), and Germany (32). Moreover, our analyses considering CPGs by country income level showed that the BBS, 6MWT, TUG, and 10mWT, are currently recommended by CPGs from middle- and high-income countries. This set of tools is similar to the consensus-based core set of outcome measures for clinical motor rehabilitation after stroke (48), which included the BBS, Fugl-Meyer Motor Assessment, 10mWT, and TUG for the lower extremity section (48). Furthermore, the BBS has also been included in a core set of recommendations for measuring standing balance in adult populations (96).

The scope of components of postural control captured by tools included in the CPGs for stroke is consistent with results of a review of standing balance measures for adult populations (69). Although some components of postural control (e.g., underlying motor systems, anticipatory postural control, and dynamic stability) are challenged in a high proportion of tools (85% or over), 41% of tools challenge a limited number (≤3) of postural control components. Conversely, less than 15% of tools recommended in these CPGs require activities that challenge reactive postural control, verticality, and cognitive influences on balance.

Previous work has highlighted the importance of reactive postural control as a predictor of future falls (97–99). The BBS, 6MWT, TUG, or 10mWT, tools most commonly recommended by CPGs in this review and in a core set for clinical motor rehabilitation after stroke (48), do not challenge cognitive influences, verticality, and reactive postural control. As a standalone tool, the Mini-BESTest is the most comprehensive, as it addresses eight components of postural control. An international panel recently recommended the Mini-BESTest, along with the BBS, for measuring standing balance in adult populations (96). Only two CPGs (72, 74), however, include the Mini-BESTest. With respect to clinical implementation, while PTs acknowledge the importance of reactive balance for function, some are hesitant to measure reactive balance in clinical practice due to perceived patient fear when they assume the leaning position required for the test, personal fear of injury, and belief that reactive balance is a higher-order skill that should only be evaluated and addressed after other components of postural control have improved (100). Continued work is needed to support recommendation and implementation of a comprehensive approach to balance assessment that includes reactive control in people post-stroke and rehabilitation more broadly.

The varied number of tools and the lack of agreement across CPGs may be due to the methods used for selecting and recommending the assessment tools (101). Authors of a previous review (101) argued that some of the variation among treatment recommendations across CPGs could be explained by the differing methods used by each guideline development group. In our review, only half of the CPGs provided information on how tools were selected. For example, three CPGs (74, 79, 86) that specified assessment tools describe conducting systematic reviews and appraisal of literature, but do not provide results of these steps for the selection of assessment tools. These findings highlight the need for improvement in the development and reporting of the methods for selecting and recommending assessment tools. Moreover, when conducting additional studies to inform the selection and recommendation of the assessment tools (102), we recommend CPGs cite these additional publications as a source for more details. The lack of description makes it difficult for guideline developers to replicate methods, and to identify the sources of variability in assessment tools recommended in CPGs for stroke (37).

A number of characteristics contribute to the feasibility of implementing the most widely recommended tools (i.e., BBS, 6MWT, TUG, or 10mWT): they have been highly recommended for use in multiple settings across the care continuum (103), they are free to use, easy to score, administration time is less than 15 min, and the tools do not require specialized training or equipment (104). In addition, versions of the BBS are available in many languages (e.g., Brazilian-Portuguese (105), English (106), German (107), Japanese (108), Norwegian (109), Persian (110), Spanish (111), Turkish (112), and Urdu (113)). Despite the availability of stroke-specific protocols for administering the 10mWT and 6MWT (114), there are challenges with their implementation in clinical practice. Some physical therapists in acute care settings view these tests as impractical as most of their patients have low levels of ambulation, and they believe that patients must be able to walk for 6 min without stopping before they administer the 6MWT (22). Organizational challenges to implementing the 10mWT and 6MWT across clinical settings relate to hospital policy against taping floors and walls to set up walkways (115), and difficulty finding space for the 30-metre walkway recommended for the 6MWT (22). Resources, such as theory-informed toolkits with implementation strategies, and onsite facilitation, can support clinical integration of standardized assessment tools (22, 116). Our findings show that, although 70% of CPGs provide resources to help clinicians implement these tools, the content of resources varies considerably, even for the administration of the same tools. For example, of the CPGs included in our review, four provided a protocol as a resource for conducting the 6MWT. One recommended a walkway of at least 12 meters (20), another recommended a 30-meter walkway (74), and two recommended a walkway of either 10, 20, 30 or 50 meters (45, 72). Moreover, the distance recommended for assessing walking speed varied and included timing a 5-, 6- or 10-meter distance. The use of different protocols, including walkway surface, length and shape, and use of walking aid and encouragement during the execution of the test, can influence the test results and limit comparisons (117–119).

A limited number of the CPGs (75, 77, 80, 81, 83, 84, 86) in this scoping review were developed in middle-income countries while none were from a low-income country, consistent with findings from a previous review (120). This suggests a limitation in the use or implementation of CGPs in the continuum of stroke care in low and middle-income countries (LMIC) (38). Previous research has outlined the challenges to development and implementation of stroke rehabilitation in LMIC (38). Most LMIC lack the human, technical and financial resources required to conduct such adaptations, let al.one develop their own CPGs. To build capacity in the global stroke rehabilitation community, Bernhardt et al. (38) have suggested a central resource of best-practice and implementation tools. Such a repository could be used by professional leaders internationally to review existing high-quality CPGs and adapt those to their local resources and context (38). Our review highlights the need for consensus on an established protocol for using these tools. We encourage guideline developers and end-users to consider these resources as they provide valuable implementation tools for commonly used and recommended tools for assessing balance and mobility.

### 4.1. Strengths and limitations

A strength of the review is the comprehensive search strategy specific to guidelines and to countries with varying income levels. The search was complemented by a grey literature search wherein CPGs were retrieved from member associations of World Physiotherapy and the World Stroke Organization. Second, we have a research team with diverse experiences, as well as diversity in language, culture, ethnicity, age and educational background. This diversity allowed for the inclusion of CPGs written in four languages, and enriched the interpretation of findings. Although we made efforts to maximize inclusion of CPGs based on language, we excluded 7 records written in languages (Chinese, Korean, Turkish, Persian and Dutch – the English version of the Dutch guideline was included) that the research team could not read. Although the majority (14/19; 74%) of included CPGs were written in English, only eight (42%) were from English-speaking countries. Findings related to the components of postural control challenged in the identified tools should be interpreted with caution. We identified the components of postural control challenged in tools that primarily focus on assessing mobility. Selecting a measure of balance that captures the components of postural control of interest is preferred, however, as the scoring for that measure is designed to reflect balance ability.

## 5. Conclusions

CPGs for stroke rehabilitation do not consistently provide recommendations for standardized tools to assess balance and mobility or resources to help end-users with clinical application. Reporting of processes for tool selection and recommendation is inadequate. Recommended assessment tools do not capture the breadth of components of postural control underlying balance and mobility. Review findings can be used to inform global efforts to develop and translate recommendations and resources for using standardized tools to assess balance and mobility post-stroke.

## Data Availability

The original contributions presented in the study are included in the article/Supplementary Material, further inquiries can be directed to the corresponding author/s.
