# Supplementary material for: Standardized tools for assessing balance and mobility in stroke clinical practice guidelines worldwide: A scoping review
Source: Front Rehabil Sci. 2023 Feb 21;4:1084085. doi: 10.3389/fresc.2023.1084085 (PMC9989207; doi:10.3389/fresc.2023.1084085)
Supplement: Supplementary file 1 [file Supplementaryfile1.docx]

**Supplementary file 1. Operational definitions**

| **Concept** | **Operational definition** |
| --- | --- |
| Clinical practice guideline | Systematically developed statements to assist practitioner decisions about appropriate health care for specific clinical circumstances (1), including recommendations on the delivery of rehabilitation (defined as recommendations related to assessment and/or treatment) of balance and/or mobility. |
| Country income level | Classified by the World Bank(2) based on gross national income (GNI) per capita in 2019:  Low-income: GNI per capita ≤ US$1,035  Lower middle-income: GNI per capita US$1,036 - $4,045  Upper middle-income: GNI per capita US$4,046 - $12,535  High-income: GNI per capita ≥ US$12,536. |
| Resource | Information (e.g., tables, links to online resources) designed to guide end-users with use (e.g., administration, interpretation) of the assessment tool. |
| Standardized assessment tool | A tool with a specific testing protocol and scoring procedure (3). |
| Tool for the performance-based assessment of balance and/or mobility | 1) Assessment tool has a stated objective or is commonly used to assess balance (defined as the ability to keep the center of mass within the base of support (4)) and/or mobility (defined as changing body position, walking and moving (adapted from the International Classification of Functioning, Disability and Health (5)) as:  a. Indicated by the guideline as a tool for assessing balance and/or mobility; or  b. Indicated in the first publication presenting its development and/or initial psychometric evaluation; and  2) Scoring is based on the performance of a balance and/or mobility balance task (i.e., excluded if self-report) as determined by the research team. |

**References**

1. Field M, Lohr K. “Attributes of good practice guidelines.,” *Clinical practice guidelines: directions for a new program*. (1990)

2. World Bank Country and Lending Groups - Country Classification. https://datahelpdesk.worldbank.org/knowledgebase/articles/906519-world-bank-country-and-lending-groups [Accessed June 13, 2021]

3. Royal College of Occupational Therapists. Research Briefing Measuring Outcomes - Nov2015.

4. Pollock AS, Durward BR, Rowe PJ, Paul JP. What is balance? *Clin Rehabil* (2000) 14: doi: 10.1191/0269215500cr342oa

5. World Health Organization. World Health Organisation. (2001). International Classification of Functioning, Disability and Health (ICF). Geneva: World Health Organisation. *International Classification* (2001) 1149:
