# Supplementary material for: Standardized tools for assessing balance and mobility in stroke clinical practice guidelines worldwide: A scoping review
Source: Front Rehabil Sci. 2023 Feb 21;4:1084085. doi: 10.3389/fresc.2023.1084085 (PMC9989207; doi:10.3389/fresc.2023.1084085)
Supplement: Supplementary file 2 [file Supplementaryfile2.docx]

**Supplementary file 2. Exemplar of search strategy used on Ovid/Medline and the hand search method to contact member associations of World Physiotherapy and the World Stroke Organization**

|  | **Database:** Ovid MEDLINE: Epub Ahead of Print, In-Process & Other Non-Indexed Citations, Ovid MEDLINE® Daily and Ovid MEDLINE® <1946-Present> |  |
| --- | --- | --- |
| **#** | **Query** | **Results from 13 Dec 2021** |
| 1 | exp Clinical Protocols/ | 180,186 |
| 2 | exp Consensus Development Conference/ or exp Consensus/ or exp Consensus Development Conferences as Topic/ | 31,588 |
| 3 | exp Critical Pathways/ | 7,314 |
| 4 | exp Practice Guideline/ or exp Guideline/ | 36,476 |
| 5 | Practice Guidelines as Topic/ | 126,200 |
| 6 | Health Planning Guidelines/ | 4,145 |
| 7 | (guideline or practice guideline or consensus development conference or consensus development conference, NIH).pt. | 46,237 |
| 8 | (position statement* or policy statement* or practice parameter* or best practice*).ti,ab,kf,kw. | 39,561 |
| 9 | (standards or guideline or guidelines).ti,kf,kw. | 122,781 |
| 10 | ((practice or treatment* or clinical) adj guideline*).ab. | 46,176 |
| 11 | (CPG or CPGs).ti. | 6,094 |
| 12 | consensus*.ti,kf,kw. | 30,395 |
| 13 | consensus*.ab. /freq=2 | 29,483 |
| 14 | ((critical or clinical or practice) adj2 (path or paths or pathway or pathways or protocol*)).ti,ab,kf,kw. | 23,363 |
| 15 | recommendat*.ti,kf,kw. | 47,429 |
| 16 | (care adj2 (standard or path or paths or pathway or pathways or map or maps or plan or plans)).ti,ab,kf,kw. | 70,674 |
| 17 | exp Stroke/ or exp Stroke Rehabilitation/ | 156,015 |
| 18 | exp "Intracranial Embolism and Thrombosis"/ | 21,958 |
| 19 | exp Intracranial Hemorrhages/ | 75,538 |
| 20 | exp Brain Ischemia/ | 116,579 |
| 21 | stroke*.tw,kf. | 282,775 |
| 22 | poststroke*.tw,kf. | 5,858 |
| 23 | (apoplex* or appoplex*).tw,kf. | 3,419 |
| 24 | ((subarachnoid or "sub arachnoid" or intracerebral or intra cerebral or subdural or "sub dural" or intracracinal or intra cranial) adj2 (hemorrhage or haemorrhage)).tw,kf. | 42,497 |
| 25 | ((cerebr* or basilar or carotid or vertebral) adj3 (occlusion* or stenos* or thrombos* or embolism or aneurysm)).tw,kf. | 58,253 |
| 26 | CVA.tw,kf. | 3,212 |
| 27 | neurologi* [condition.tw](http://condition.tw/),kf. | 3,033 |
| 28 | 1 or 2 or 3 or 4 or 5 or 6 or 7 or 8 or 9 or 10 or 11 or 12 or 13 or 14 or 15 or 16 | 637,324 |
| 29 | 17 or 18 or 19 or 20 or 21 or 22 or 23 or 24 or 25 or 26 or 27 | 466,453 |
| 30 | 28 and 29 | 12,263 |
| 31 | limit 30 to yr="2014 - 2021" | 6,458 |

| **Steps** | **Detail** |
| --- | --- |
| 1. Country |  |
| 1. Association/Organization/Member |  |
| 1. E-mail address | Send the “Initial” e-mail preferably on a Tuesday  If no email is provided on the website, write ‘**NP**’ and go to step 16 |
| 1. Contact date | (dd/mm/yyyy) |
| 1. Response (Yes/No) | If Yes, go to Step 17  If No, go to Step 6 |
| 1. 1^st^ Reminder | Send 1^st^ reminder **2 days** after initial e-mail |
| 1. Response (Yes/No) | If Yes, go to Step 17  If No, go to Step 8 |
| 1. 2^nd^ Reminder | Send 2^nd^ reminder **7 days** after the 1^st^ reminder |
| 1. Response (Yes/No) | If Yes, go to Step 17  If No, go to Step 16 |
| 1. Contact Expert Group E-mail |  |
| 1. Response (Yes/No) | If Yes, go to Step 17  If No, go to Step 12 |
| 1. 1^st^ Reminder | Send 1^st^ reminder **2 days** after initial e-mail |
| 1. Response (Yes/No) | If Yes, go to Step 17  If No, go to Step 14 |
| 1. 2^nd^ Reminder | Send 2^nd^ reminder **7 days** after the 1^st^ reminder |
| 1. Response (Yes/No) | If Yes, go to Step 17  If No, go to Step 16 |
| 1. Manually Search Website | After manually searching the website, go to step 17 |
| 1. Guideline (Yes/No) | Full name |
| 1. Year | YYYY |
| 1. Language |  |

| **Email Template** |
| --- |
| **INITIAL E-MAIL**  Subject: Request for Information on Stroke Rehabilitation Guidelines  Dear XXXXX,  My name is XXXX, and I am a physiotherapy student at XXXXX.  I am writing to you as your name appears as the contact of the Physiotherapy Association of your country on the World Physiotherapy website (<https://world.physio/our-members>).  I am conducting a scoping review of existing stroke rehabilitation guidelines worldwide, supervised by Dr. Nancy Salbach (University of Toronto, Canada), Dr. Gudrun Diermayr (SRH Hochschule Heidelberg, Germany), and Dr. Kathryn Sibley (University of Manitoba, Canada).  We aim to identify which stroke guidelines include recommendations and resources for the use of standardized assessment tools. We are specifically interested in:   - stroke rehabilitation guidelines, - stroke physiotherapy clinical practice guidelines or - neurological rehabilitation guidelines that apply to stroke.   Does your physiotherapy association or country have a stroke rehabilitation guideline?  If yes, we would be grateful if you could share the PDF file of the guideline or the link to the guideline.  If you are unsure, would you be able to refer us to someone that could provide the guideline, the link to or information about stroke guidelines in your country?    Thank you for your time.    Sincerely,  XXXXXX  **1^st^ REMINDER**  Dear xxx, this is a friendly reminder about our inquiry below. Please let us know if you have any questions and thank you for your time.  **2^nd^ REMINDER**  Next and final reminder: Dear xxx, this is a final reminder about our inquiry below. Please let us know if you have any questions and thank you for your time |
