# Supplementary material for: Standardized tools for assessing balance and mobility in stroke clinical practice guidelines worldwide: A scoping review
Source: Front Rehabil Sci. 2023 Feb 21;4:1084085. doi: 10.3389/fresc.2023.1084085 (PMC9989207; doi:10.3389/fresc.2023.1084085)
Supplement: Supplementary file 3 [file Supplementaryfile3.docx]

**Supplementary file 3. Assessment tools and resources included in stroke clinical practice guidelines worldwide**

| **Guideline: *Accident vasculaire cérébral Pertinence des parcours de rééducation/réadaptation après la phase initiale de l’AVC - Note de Problématique***(1) | | | | |
| --- | --- | --- | --- | --- |
| **Name and/or version of the assessment tool** | **Level of recommendation** | **Construct assessed according to the guideline** | **Time of administration recommended by the guideline** | **Resources** |
| Action Research Arm Test (ARAT) | Not reported | UE motor function | Not reported | Not reported |
| Barthel Index of Activities of Daily Living (BI) | Not reported | Global functional deficit, Autonomy, ADL | Not reported | Not reported |
| Batterie d’évaluation de la négligence spatiale unilatérale du GEREN (BEN) | Not reported | Hemineglect | Not reported | Not reported |
| **Berg Balance Scale (BBS)** | Not reported | Balance in seated and standing position | Not reported | Page 18: <https://www.has-sante.fr/upload/docs/application/pdf/Evaluation_%20fonctionnelle_%20AVC_ref.pdf>  Page 48: <https://publications.msss.gouv.qc.ca/msss/fichiers/2017/17-944-03W.pdf> |
| Box and Block Test (BBT) | Not reported | Dexterity | Not reported | Page 55: <https://publications.msss.gouv.qc.ca/msss/fichiers/2017/17-944-03W.pdf> |
| Canadian Neurological Scale (CNS) | Not reported | Stroke severity | Not reported | Not reported |
| Chedoke Arm and Hand Activity Inventory-9 (CAHAI-9) | Not reported | UE motor function | Not reported | Page 54: <https://publications.msss.gouv.qc.ca/msss/fichiers/2017/17-944-03W.pdf> |
| **Chedoke-McMaster Stroke Assessment (CMSA)** | Not reported | Ability to change position, Standing balance | Not reported | Page 35: <https://publications.msss.gouv.qc.ca/msss/fichiers/2017/17-944-03W.pdf> |
| Chedoke-McMaster Stroke Assessment – Activity inventory (CMSA-AI) | Not reported | Functional autonomy | Not reported | Not reported |
| Chedoke-McMaster Stroke Assessment – Impairment inventory (CMSA-II) | Not reported | Arm, leg, foot motor function, Functional autonomy | Not reported | Page 40: <https://publications.msss.gouv.qc.ca/msss/fichiers/2017/17-944-03W.pdf> |
| la cotation de Held et Pierrot-Deseilligny | Not reported | Voluntary motricity | Not reported | Page 25: <https://www.has-sante.fr/upload/docs/application/pdf/Evaluation_%20fonctionnelle_%20AVC_ref.pdf> |
| Daniels and Worthingham method | Not reported | Voluntary motricity | Not reported | Page 38: <https://publications.msss.gouv.qc.ca/msss/fichiers/2017/17-944-03W.pdf> |
| Demeurisse Motricity Index | Not reported | Motricity | Not reported | Page 28: <https://www.has-sante.fr/upload/docs/application/pdf/Evaluation_%20fonctionnelle_%20AVC_ref.pdf> |
| l’échelle Catherine Bergego (ECB) | Not reported | Hemineglect impact in ADL | Not reported | Page 20: <https://www.has-sante.fr/upload/docs/application/pdf/Evaluation_%20fonctionnelle_%20AVC_ref.pdf> |
| l’échelle neurologique canadienne (ENC) | Not reported | Stroke severity | Not reported | Not reported |
| Erasmus Nottingham Sensory Assessment (EmNSA) | Not reported | Sensibility | Not reported | Page 36: <https://publications.msss.gouv.qc.ca/msss/fichiers/2017/17-944-03W.pdf> |
| Frenchay Activity Index | Not reported | Global functional deficit, Autonomy, UE motor function, ADL | Not reported | Page 26: <https://www.has-sante.fr/upload/docs/application/pdf/Evaluation_%20fonctionnelle_%20AVC_ref.pdf> |
| Functional Autonomy Measurement System (SMAF) | Not reported | Functional autonomy | Not reported | Page 43: <https://publications.msss.gouv.qc.ca/msss/fichiers/2017/17-944-03W.pdf> |
| Functional Independence Measure (FIM) | Not reported | Global functional deficit, autonomy, ADL | Not reported | Not reported |
| Fugl-Meyer Assessment of Motor Recovery after Stroke (FMA) | Not reported | Motricity | Not reported | Not reported |
| General Health Questionnaire (GHQ) | Not reported | Participation | Not reported | Not reported |
| Glasgow | Not reported | Stroke severity | Not reported | Not reported |
| Hospital Anxiety and Depression Scale (HADS) | Not reported | Psychoaffective disorders | Not reported | Page 22: <https://publications.msss.gouv.qc.ca/msss/fichiers/2017/17-944-03W.pdf> |
| ***l’indice d’équilibre postural assis* (EPA)** | Not reported | Balance in seated position | Not reported | Page 30: <https://www.has-sante.fr/upload/docs/application/pdf/Evaluation_%20fonctionnelle_%20AVC_ref.pdf> |
| ***l’indice d’équilibre postural debout* (EPD)** | Not reported | Standing balance | Not reported | Page 31: <https://www.has-sante.fr/upload/docs/application/pdf/Evaluation_%20fonctionnelle_%20AVC_ref.pdf> |
| Manual dinamometry | Not reported | Grip strength | Not reported | Not reported |
| Medical Outcome Study Short Form 36 (SF-36) | Not reported | Participation | Not reported | Not reported |
| Mayo Portland Adaptability Inventory-4 (MPAI-4), partie C, Participation | Not reported | Participation | Not reported | Page 66: <https://publications.msss.gouv.qc.ca/msss/fichiers/2017/17-944-03W.pdf> |
| **Mini-BESTest** | Not reported | Standing balance | Not reported | Page 49: <https://publications.msss.gouv.qc.ca/msss/fichiers/2017/17-944-03W.pdf> |
| Modified Ashworth Scale (MAS) | Not reported | Tone | Not reported | Page 37: <https://publications.msss.gouv.qc.ca/msss/fichiers/2017/17-944-03W.pdf>  Page 17: <https://www.has-sante.fr/upload/docs/application/pdf/Evaluation_%20fonctionnelle_%20AVC_ref.pdf> |
| Modified Rankin Scale | Not reported | Global functional deficit, autonomy | Not reported | Page 39: <https://www.has-sante.fr/upload/docs/application/pdf/Evaluation_%20fonctionnelle_%20AVC_ref.pdf> |
| Montreal Cognitive Assessment (MoCA©) | Not reported | Cognitive function | Not reported | Page 26: <https://publications.msss.gouv.qc.ca/msss/fichiers/2017/17-944-03W.pdf> |
| **Motor Assessment Scale (MAS)** | Not reported | Ability to change position, Balance in seated position, Gait performance | Not reported | Page 34: <https://www.has-sante.fr/upload/docs/application/pdf/Evaluation_%20fonctionnelle_%20AVC_ref.pdf> |
| Motor-Free Visual Perception Test (MVPT) | Not reported | Vision | Not reported | Page 33: <https://publications.msss.gouv.qc.ca/msss/fichiers/2017/17-944-03W.pdf> |
| National Institutes of Health Stroke Scale (NIHSS) | Not reported | Stroke severity | Not reported | Not reported |
| Neurobehavioral Cognitive Status Examination (NCSE ou Cognistat) | Not reported | Cognitive function | Not reported | Not reported |
| Nine Hole Peg Test | Not reported | Dexterity | Not reported | Page 56: <https://publications.msss.gouv.qc.ca/msss/fichiers/2017/17-944-03W.pdf> |
| Nottingham Extended Activities of Daily Living (NEADL) Scale | Not reported | Participation | Not reported | Not reported |
| Nottingham Health Profile (NHP) | Not reported | Participation | Not reported | Not reported |
| Orgogozo Scale | Not reported | Motricity | Not reported | Page 23: <https://www.has-sante.fr/upload/docs/application/pdf/Evaluation_%20fonctionnelle_%20AVC_ref.pdf> |
| **Performance Oriented Mobility Assessment**  **(POMA)** | Not reported | Standing balance | Not reported | Not reported |
| **Postural Assessment Scale for Stroke Patients (PASS)** | Not reported | Ability to change position, Balance in seated and standing position | Not reported | Page 37: <https://www.has-sante.fr/upload/docs/application/pdf/Evaluation_%20fonctionnelle_%20AVC_ref.pdf> |
| Questionnaire sur la santé du patient (QSP-9) | Not reported | Psychoaffective disorders | Not reported | Page 23: <https://publications.msss.gouv.qc.ca/msss/fichiers/2017/17-944-03W.pdf> |
| Rankin Handicap Scale | Not reported | ADL | Not reported | Page 39: <https://www.has-sante.fr/upload/docs/application/pdf/Evaluation_%20fonctionnelle_%20AVC_ref.pdf> |
| **Rivermead Motor Assessment (RMA)** | Not reported | Ability to change position, ADL | Not reported | Page 42: <https://www.has-sante.fr/upload/docs/application/pdf/Evaluation_%20fonctionnelle_%20AVC_ref.pdf> |
| Rivermead Mobility Index (RMI) | Not reported | Ability to change position | Not reported | Page 41: <https://www.has-sante.fr/upload/docs/application/pdf/Evaluation_%20fonctionnelle_%20AVC_ref.pdf> |
| Sickness Impact Profile (SIP) (Stroke-Adapted Version) or Stroke-Adapted Sickness Impact Profile | Not reported | Participation | Not reported | Not reported |
| **Sødring Motor Evaluation of Stroke Patients (SMES)** | Not reported | Motricity, UE function | Not reported | Page 43: <https://www.has-sante.fr/upload/docs/application/pdf/Evaluation_%20fonctionnelle_%20AVC_ref.pdf> |
| **Step Test** | Not reported | Standing balance | Not reported | Page 45: <https://www.has-sante.fr/upload/docs/application/pdf/Evaluation_%20fonctionnelle_%20AVC_ref.pdf> |
| Stroke Aphasic Depression Questionnaire (SADQH-10 ou SADQ-10) | Not reported | Psychoaffective disorders for people with aphasia | Not reported | Page 24: <https://publications.msss.gouv.qc.ca/msss/fichiers/2017/17-944-03W.pdf> |
| Stroke Impact Scale (SIS) | Not reported | ADL | Not reported | Page 46: <https://www.has-sante.fr/upload/docs/application/pdf/Evaluation_%20fonctionnelle_%20AVC_ref.pdf> |
| **Stroke Rehabilitation Assessment of Movement (STREAM)** | Not reported | Motricity, Ability to change position | Not reported | Page 47: <https://www.has-sante.fr/upload/docs/application/pdf/Evaluation_%20fonctionnelle_%20AVC_ref.pdf> |
| Stroke-Specific Geriatric Depression Scale (SS-GDS) | Not reported | Psychoaffective disorders for older people | Not reported | Page 25: <https://publications.msss.gouv.qc.ca/msss/fichiers/2017/17-944-03W.pdf> |
| Stroke-Specific Quality of Life Scale (SS-QOL) | Not reported | Participation | Not reported | Not reported |
| Tardieu Scale | Not reported | Tone | Not reported | Page 24: <https://www.has-sante.fr/upload/docs/application/pdf/Evaluation_%20fonctionnelle_%20AVC_ref.pdf> |
| **Timed Up and Go Test (TUG)** | Not reported | Gait performance | Not reported | Page 54: <https://www.has-sante.fr/upload/docs/application/pdf/Evaluation_%20fonctionnelle_%20AVC_ref.pdf> |
| **Trunk Control Test (TCT)** | Not reported | Balance in seated position | Not reported | Page 55: <https://www.has-sante.fr/upload/docs/application/pdf/Evaluation_%20fonctionnelle_%20AVC_ref.pdf> |
| **6-Minute Walk Test (6MWT)** | Not reported | Gait performance | Not reported | Page 53: <https://www.has-sante.fr/upload/docs/application/pdf/Evaluation_%20fonctionnelle_%20AVC_ref.pdf> |
| **10-Meter Walk Test (10mWT)** | Not reported | Gait performance | Not reported | Page 52: <https://www.has-sante.fr/upload/docs/application/pdf/Evaluation_%20fonctionnelle_%20AVC_ref.pdf> |

Abbreviations: UE: upper extremity; ADL: activities of daily living

| **Guideline: A core set of outcome measures for adults with neurologic conditions undergoing rehabilitation**(2) | | | | |
| --- | --- | --- | --- | --- |
| **Name and/or version of the assessment tool** | **Level of recommendation^*^** | **Construct assessed according to the guideline** | **Time of administration recommended by the guideline** | **Resources** |
| Activities-specific Balance Confidence (ABC) Scale | Acute conditions: Evidence quality: I; recommendation strength: strong. Chronic stable conditions: Evidence quality: I; recommendation strength: strong. Chronic progressive conditions: Evidence quality: I; recommendation strength: strong | Self-reported balance confidence | At admission, and discharge, and when feasible, between these periods for acute and chronic conditions^†^ | <https://www.neuropt.org/docs/default-source/cpgs/core-outcome-measures/core-measure-activities-specific-balance-confidence-scale-(abc-scale)_final-2020af1837a5390366a68a96ff00001fc240.pdf?sfvrsn=6f1b5143_0>  https://www.neuropt.org/docs/default-source/cpgs/core-outcome-measures/activities-specific-balance-confidence-scale-proof8-(2)17db36a5390366a68a96ff00001fc240.pdf?sfvrsn=d7d85043_0  https://www.neuropt.org/docs/default-source/cpgs/core-outcome-measures/kt-report-card-simplified-2021_2.pdf?sfvrsn=19ae5f43_0 |
| **Berg Balance Scale (BBS)** | Acute conditions: Evidence quality: I; recommendation strength: strong. Chronic stable conditions: Evidence quality: I; recommendation strength: strong. Chronic progressive conditions: Evidence quality: I; recommendation strength: strong | Static and dynamic sitting, and standing balance | At admission, and discharge, and when feasible, between these periods for acute and chronic conditions | https://www.neuropt.org/docs/default-source/cpgs/core-outcome-measures/berg-balance-scale-pocket-guide-proof-8.pdf?sfvrsn=8fe25043_0  https://www.neuropt.org/docs/default-source/cpgs/core-outcome-measures/core-measure-berg-balance-scale-(bbs)_final-2019.pdf?sfvrsn=6e845043_0 https://www.neuropt.org/docs/default-source/cpgs/core-outcome-measures/kt-task-force-update-sheet30b536a5390366a68a96ff00001fc240.pdf?sfvrsn=ecb65043_0  https://www.neuropt.org/docs/default-source/cpgs/core-outcome-measures/kt-report-card-simplified-2021_2.pdf?sfvrsn=19ae5f43_0 |
| **Functional Gait Assessment (FGA)** | Acute conditions: Evidence quality: I; recommendation strength: strong. Chronic stable conditions: Evidence quality: I; recommendation strength: strong. Chronic progressive conditions: Evidence quality: I; recommendation strength: moderate | Walking balance | At admission, and discharge, and when feasible, between these periods for acute and chronic conditions | <https://www.neuropt.org/docs/default-source/cpgs/core-outcome-measures/core-measure-functional-gait-assessment_final.pdf?sfvrsn=d4585243_2&sfvrsn=d4585243_2>  <https://www.neuropt.org/docs/default-source/cpgs/core-outcome-measures/function-gait-assessment-pocket-guide-proof9-(2).pdf?sfvrsn=b4f35043_0>  <https://www.neuropt.org/docs/default-source/cpgs/core-outcome-measures/kt-task-force-update-sheet30b536a5390366a68a96ff00001fc240.pdf?sfvrsn=ecb65043_0>  <https://neuropt.org/docs/default-source/cpgs/core-outcome-measures/environmental-set-up-for-core-measures-administration-tips-for-success.pdf?sfvrsn=81f5343_2&sfvrsn=81f5343_2> |
| Goal Attainment Scale (GAS) | Evidence quality: V; recommendation strength: best practice | Patient-stated goals | At least 2 times, at admission and discharge, and, when feasible, between these testing periods |  |
| **5-Times Sit-to-Stand (5TSTS)** | Evidence quality: V; recommendation strength: best practice | Transfers | At admission, and discharge, and when feasible, between these periods for acute and chronic conditions | <https://www.neuropt.org/docs/default-source/cpgs/core-outcome-measures/core-measure-five-times-sit-to-stand-(5tsts)_final.pdf?sfvrsn=dc585243_2&sfvrsn=dc585243_2>  <https://www.neuropt.org/docs/default-source/cpgs/core-outcome-measures/5tsts-pocket-guide-v2-proof9-(2)38db36a5390366a68a96ff00001fc240.pdf?sfvrsn=f4d85043_0>  https://www.neuropt.org/docs/default-source/cpgs/core-outcome-measures/kt-report-card-simplified-2021_2.pdf?sfvrsn=19ae5f43_0 |
| **6-Minute Walk Test (6MWT)** | Acute conditions: Evidence quality: V; recommendation strength: best practice. Chronic stable conditions: Evidence quality: I; recommendation strength: moderate. Chronic progressive conditions: Evidence quality: I; recommendation strength: strong | Walking distance | At admission, and discharge, and when feasible, between these periods for acute and chronic conditions | https://neuropt.org/docs/default-source/cpgs/core-outcome-measures/core-outcome-measures-documents-july-2018/6mwt_protocol.pdf?sfvrsn=fc325343_2&sfvrsn=fc325343_2  <https://www.neuropt.org/docs/default-source/cpgs/core-outcome-measures/6mwt-pocket-guide-proof9.pdf?sfvrsn=9ee25043_0>  <https://www.neuropt.org/docs/default-source/cpgs/core-outcome-measures/kt-report-card-simplified-2021_2.pdf?sfvrsn=19ae5f43_0>  https://neuropt.org/docs/default-source/cpgs/core-outcome-measures/environmental-set-up-for-core-measures-administration-tips-for-success.pdf?sfvrsn=81f5343_2&sfvrsn=81f5343_2 |
| **10-Meter Walk Test (10mWT)**  (6-meter distance is timed) | Acute conditions: Evidence quality: V; recommendation strength: best practice. Chronic stable conditions: Evidence quality: I; recommendation strength: strong. Chronic progressive conditions: Evidence quality: I; recommendation strength: strong | Walking speed | At admission, and discharge, and when feasible, between these periods for acute and chronic conditions | https://www.neuropt.org/docs/default-source/cpgs/core-outcome-measures/core-measure-10-meter-walk-test-(10mwt)_final.pdf?sfvrsn=c5585243_2&sfvrsn=c5585243_2  <https://www.neuropt.org/docs/default-source/cpgs/core-outcome-measures/10mwt-pocket-guide-rev-0520.pdf?sfvrsn=90145143_0>  <https://www.neuropt.org/docs/default-source/cpgs/core-outcome-measures/kt-report-card-simplified-2021_2.pdf?sfvrsn=19ae5f43_0>  https://neuropt.org/docs/default-source/cpgs/core-outcome-measures/environmental-set-up-for-core-measures-administration-tips-for-success.pdf?sfvrsn=81f5343_2&sfvrsn=81f5343_2 |

^*^Evidence quality I: evidence obtained from at least one high-quality (>50% critical appraisal score) study of psychometric properties; Evidence quality V: expert opinion (or best practice). Recommendation strength - Strong: a preponderance of level I studies, but least 1 level I study directly on the topic supports the recommendation; Recommendation strength - Moderate: A preponderance of level II studies, but at least 1 level II study directly on the topic supports the recommendation; Recommendation strength – Best practice: best practice based on expert opinion (review papers, white papers, consensus documents) developed by various methodologies (e.g., Delphi and RAND) and the clinical experience of the guideline development group.

^†^Acute: less than 6 months; Chronic stable: more than 6 months but not expected to progress; Chronic progressive conditions: more than 6 months with potential to experience additional symptoms or functional decline (not applicable to stroke)

| **Guideline:**  **Canadian Stroke Best Practices Recommendations - Rehabilitation and Recovery following Stroke - 6th Edition - 2019 updated**(3) | | | | |
| --- | --- | --- | --- | --- |
| **Name and/or version of the assessment tool** | **Level of recommendation** | **Construct assessed according to the guideline** | **Time of administration recommended by the guideline** | **Resources** |
| ABILHAND | Not reported | Tool for performing bimanual ADL - functional capacity and ADL | Not reported | The measure and its corresponding analysis can be viewed for free at: http://rssandbox.iescagilly.be/abilhand-rasch-analysis-chronic-stroke.html |
| Action Research Arm Test (ARAT) | Not reported | UE function and dexterity | Not reported | Free: http://www.strokengine.ca/?s=action+research+arm+test |
| AlphaFIM® Instrument | Not reported | For use during acute care - Functional Capacity and ADL | Not reported | Available for purchase. www.udsmr.org/WebModules/Alpha/Alp_About.aspx https://www.udsmr.org/ |
| Barthel Index of Activities of Daily Living (BI) | Not reported | Independence in self-care activities - Functional Capacity and ADL | Not reported | http://www.strokecenter.org/wp-content/uploads/2011/08/barthel.pdf |
| Beck Depression Inventory (BDI) | Not reported | Screening tool for depression and, if present, provides cut points for severity - Mood/Cognition | Not reported | Free: http://www.strokengine.ca/?s=beck+depression+inventory |
| Behavioral Inattention Test (BIT) | Not reported | Screening and assessment tool for visual neglect - Visual Perception/Neglect | Not reported | Available for purchase: http://www.pearsonassess.ca/en/programs/00/51/95/p005195.html?CS_Category=%26CS_Catalog=TPC-CACatalog%26CS_ProductID=749129972 |
| **Berg Balance Scale (BBS)** | Not reported | Balance - Mobility | Not reported | http://www.strokengine.ca/assess/bbs/ http://strokengine.ca/assess/module_bbs_intro-en.html |
| Box and Block Test (BBT) | Not reported | Unilateral gross manual dexterity - UE | Not reported | Standardized equipment available for purchase: http://www.pattersonmedical.com/app.aspx?cmd=getProductDetail&key=070_921018701 |
| Canadian Neurological Scale (CNS) | Not reported | Neurological impairment - Stroke Severity | Not reported | Free: www.strokecenter.org/wp-content/uploads/2011/08/canadian.pdf |
| Canadian Occupational Performance Measure (COPM) | Not reported | Measures a client’s everyday functioning in self-care, productivity and leisure | Not reported | Available for purchase. http://www.thecopm.ca/buy/ |
| Chedoke Arm and Hand Activity Inventory (CAHAI) | Not reported | Arm and hand function - UE | Not reported | Free: http://www.cahai.ca/ |
| Chedoke-McMaster Stroke Assessment Scale (CMSA) | Not reported | Physical impairment and disability - Motor function | Not reported | http://www.rehabmeasures.org/PDF%20Library/CMSA%20Manual%20and%20Score%20Form.pdf https://www.sralab.org/rehabilitation-measures/chedoke-mcmaster-stroke-assessment-measure |
| **Clinical Outcome Variables Scale (COVS)** | Not reported | Functional mobility - Mobility | Not reported | http://www.irrd.ca/covs/ |
| Clock Drawing Test (CDT) | Not reported | Screening tool for cognitive impairment - Mood/Cognition | Not reported | Free: http://www.strokengine.ca/?s=clock+drawing |
| Disability Assessment Scale (DAS) | Not reported | Upper limb spasticity | Not reported | Information about the scale can be seen in the following publication by Brashear et al. 2002: https://www.ncbi.nlm.nih.gov/pubmed/12370866 |
| Frenchay Activities Index (FAI) | Not reported | Instrumental ADL - Functional Capacity and ADL | Not reported | www.rehabmeasures.org/PDF%20Library/Frenchay%20Activities%20Index.pdf |
| Fugl-Meyer Assessment of Motor Recovery after Stroke (FMA) | Not reported | Motor functioning | Not reported | http://www.rehabmeasures.org/lists/rehabmeasures/dispform.aspx?ID=908 http://strokengine.ca/assess/module_fma_intro-en.html |
| Functional Ambulation Categories (FAC) | Not reported | Rating ambulation status - Mobility | Not reported | http://www.strokengine.ca/s=functional+ambulation+categories  http://strokengine.ca/assess/module_fac_intro-en.html |
| Functional autonomy measurement system (SMAF) | Not reported | Functional independence - Functional Capacity and ADL | Not reported | Available for purchase: http://www.demarchesmaf.com/en/ |
| Functional Independence Measure (FIM) | Not reported | Tool for physical and cognitive disability and is intended to measure burden of care - Functional Capacity and ADL | Not reported | Available for purchase. www.udsmr.org/WebModules/FIM/Fim_About.aspx http://www.strokengine.ca/assess/fim/ |
| **Functional Reach Test (FRT)** | Not reported | Static balance assessing the maximum distance a participant can reach forward while standing in a fixed position - Mobility | Not reported | https://www.sralab.org/sites/default/files/2017-06/5Hgjkv-Functional%20Reach%20Test.pdf |
| General Health Questionnaire (GHQ) | Not reported | Screening tool for psychiatric disorders - Mood/Cognition | Not reported | Available for purchase: https://shop.psych.acer.edu.au/acer-shop/group/SD |
| Geriatric Depression Scale (GDS) | Not reported | Screening tool for depression and, if present, provides cut points for severity - Mood/Cognition | Not reported | Free: http://www.strokengine.ca/?s=geriatric+depression+scale |
| Hospital Anxiety and Depression Scale (HADS) | Not reported | Screening tool for anxiety and depression and, if present, provides cut points for severity - Mood/Cognition | Not reported | Available for purchase: http://www.gl-assessment.co.uk/products/hospital-anxiety-and-depression-scale-0 |
| Life Habits (LIFE-H) | Not reported | Tool for quality of social participation based on the ability to accomplish ADL and social roles - Functional Capacity and ADL | Not reported | A copy of the LIFE-H can be ordered from the International Network on the Disability Creation Process (INDCP) by emailing the coordinator at francis.charrier@idrpq.qc.ca. |
| Line Bisection Test (LBT) | Not reported | Screening tool for unilateral spatial neglect - Visual Perception/Neglect | Not reported | Available for purchase: http://www.pearsonassess.ca/en/programs/00/51/95/p005195.html?CS_Category=%26CS_Catalog=TPC-CACatalog%26CS_ProductID=749129972 |
| **Mini-BESTest** | Not reported | Balance control - Mobility | Not reported | http://www.bestest.us/ |
| Mini-Mental State Examination (MMSE) | Not reported | Screening tool for cognitive impairment - Mood/Cognition | Not reported | Available for purchase: http://www4.parinc.com/Products/Product.aspx?ProductID=MMSE |
| Modified Ashworth Scale (MAS) | Not reported | Spasticity | Not reported | Free: http://www.strokengine.ca/?s=modified+ashworth http://strokengine.ca/assess/module_mashs_intro-en.html |
| Modified Barthel Index of Activities of Daily Living (MBI) | Not reported | ADL | Not reported | http://www.strokecenter.org/trials/scales/barthel.pdf |
| Modified Rankin Scale (MRS) | Not reported | Tool for rating global outcome - Functional Capacity and ADL | Not reported | Free: www.rankinscale.org/ |
| Modified Tardieu Scale (MTS) | Not reported | Scale for spasticity in various neurological conditions | Not reported | Information about the scale can be seen in the following publication by Ansari et al., 2008: https://www.ncbi.nlm.nih.gov/pubmed/19117179 |
| Montreal Cognitive Assessment (MoCA©) | Not reported | Screening tool for cognitive impairment - Mood/Cognition | Not reported | Free: http://www.mocatest.org/ |
| Motor-free Visual Perception Test (MVPT) | Not reported | Visual perception - Visual Perception/Neglect | Not reported | Available for purchase: http://www.academictherapy.com/detailATP.tpl?action=search&cart=14301685755462655&eqskudatarq=8962-9&eqTitledatarq=Motor-Free%20Visual%20Perception%20Test-4%20%28MVPT-4%29&eqvendordatarq=ATP&bobby=%5Bbobby%5D&bob=%5Bbob%5D&TBL=[tbl |
| National Institutes of Health Stroke Scale (NIHSS) | Not reported | Neurological status following a stroke - Stroke Severity | Not reported | Free: www.strokecenter.org/wp-content/uploads/2011/08/NIH_Stroke_Scale.pdf |
| Nine Hole Peg Test (NHPT) | Not reported | Manual dexterity. - UE | Not reported | Standardized equipment available for purchase: http://www.pattersonmedical.com/app.aspx?cmd=getProduct&key=IF_921029571 |
| Orpington Prognostic Scale (OPS) | Not reported | Beneficial in identifying a patient’s suitability for rehabilitation - Stroke severity | Not reported | Free: www.uwhealth.org/files/uwhealth/docs/pdf/spep_orpington_scale.pdf |
| Patient Health Questionnaire-9 (PHQ-9) | Not reported | Screening tool for depression and provides an assessment of symptom severity as well - Mood/Cognition | Not reported | Free https://www.phqscreeners.com/ |
| Rivermead Mobility Index (RMI) | Not reported | Functional mobility - Mobility | Not reported | http://www.strokengine.ca/?s=rivermead |
| **Rivermead Motor Assessment (RMA)** | Not reported | Motor performance | Not reported | www.strokengine.ca/assess/rma/ |
| **Stroke Rehabilitation Assessment of Movement (STREAM)** | Not reported | Motor functioning | Not reported | http://ptjournal.apta.org/content/79/1/8.full.pdf+html |
| **Timed Up and Go Test (TUG)** | Not reported | Basic mobility and balance - Mobility | Not reported | [http://www.strokengine.ca/?s=timed+up+and+go http://strokengine.ca/assess/module_tug_family-en.html](http://www.strokengine.ca/?s=timed+up+and+go) |
| Wolf Motor Function Test (WMFT) | Not reported | Upper extremity motor ability - UE | Not reported | Free: http://www.strokengine.ca/?s=wolf+motor+function+test |
| **6-Minute Walk Test (6MWT)** | Not reported | Walking capacity and endurance - Functional Capacity and ADL | Not reported | The iWalk Toolkit has stroke-specific protocols, educational videos, and the iWalkAssess app. www.iwalkassess.com. http://strokengine.ca/assess/module_6mwt_intro-en.html |
| **10-Meter Walk Test (10mWT)** | Not reported | Walking speed - Functional Capacity and ADL | Not reported | http://www.rehabmeasures.org/PDF%20Library/10%20Meter%20Walk%20Test%20Instructions.pdf The iWalk Toolkit has stroke-specific protocols, educational videos, and the iWalkAssess app. |

Abbreviations: ADL: activities of daily living; UE: upper extremity

| **Guideline: DEGAM Leitlinie-S3: Schlaganfall**(4) | | | | |
| --- | --- | --- | --- | --- |
| **Name and/or version of the assessment tool** | **Level of recommendation** | **Construct assessed according to the guideline** | **Time of administration recommended by the guideline** | **Resources** |
| Barthel Index of Activities of Daily Living (BI) | Not reported | Not reported | Not reported | Not reported |
| **Timed Up and Go Test (TUG)** | Not reported | Not reported | Not reported | Not reported |
| **6-Minute Walk Test (6MWT)** | Not reported | Not reported | Not reported | Not reported |

| **Guideline: Guía de práctica clínica fisioterapéutica para la evaluación y tratamiento de pacientes con enfermedades cerebrovasculares en los primeros seis meses de la enfermedad**(5) | | | | |
| --- | --- | --- | --- | --- |
| **Name and/or version of the assessment tool** | **Level of recommendation** | **Construct assessed according to the guideline** | **Time of administration recommended by the guideline** | **Resources** |
| Action Research Arm Test (ARAT) | Not reported | Motor function of the UE | First six months | Not reported |
| Ashworth Scale | Not reported | Motor function of the UE/LE | First six months | Not reported |
| Barthel Index of Activities of Daily Living (BI) | Not reported | Motor function of the UE/LE | First six months | Not reported |
| **Berg Balance Scale (BBS)** | Not reported | Balance | First six months | Not reported |
| Box and Block Test (BBT) | Not reported | Motor function of the UL | First six months | Not reported |
| Fugl-Meyer Assessment of Motor Recovery after Stroke (FMA) | Not reported | Motor function of the UE/LE | First six months | Barbosa, N. E., Forero, S. M., Galeano, C. P., Hernández, E. D., Landinez, N. S., Sunnerhagen, K. S., & Alt Murphy, M. (2019). Translation and cultural validation of clinical observational scales - the Fugl-Meyer assessment for post stroke sensorimotor function in Colombian Spanish. Disability and rehabilitation, 41(19), 2317–2323. https://doi.org/10.1080/09638288.2018.1464604 |
| **Fugl-Meyer Balance Test** | Not reported | Balance | First six months | Not reported |
| Functional Independence Measure (FIM) | Not reported | Selfcare | First six months | Not reported |
| **Functional Reach Test (FRT)** | Not reported | Balance | First six months | Not reported |
| Grip strength | Not reported | Motor function of the UE | First six months | Not reported |
| Lawton modified | Not reported | Instrumental ADL | First six months | Not reported |
| Motor Activity Log | Not reported | Motor function of the UE | First six months | Not reported |
| Motricity Index | Not reported | Motor function of the LE | First six months | Not reported |
| **Performance Oriented Mobility Assessment (POMA)** | Not reported | Balance | First six months | Not reported |
| **Rivermead Motor Assessment (RMA)** | Not reported | Balance | First six months | Not reported |
| **Timed Up and Go Test (TUG)** | Not reported | Balance | First six months | Not reported |

Abbreviations: UE: upper extremity; LE: lower extremity; ADL: activities of daily living

| **Guideline: Guidelines for Adult Stroke Rehabilitation and Recovery: A Guideline for Healthcare Professionals from the American Heart Association/American Stroke Association**(6) | | | | |
| --- | --- | --- | --- | --- |
| **Name and/or version of the assessment tool** | **Level of recommendation** | **Construct assessed according to the guideline** | **Time of administration recommended by the guideline** | **Resources** |
| Action Research Arm Test (ARAT) | Not reported | UE function | Not reported | Criteria based with 19 items; scores are from 0–57, with normal=57; allows observation of multiple grasps, grips, and pinches. Time to administer (min.): 10 |
| Activities-specific Balance Confidence (ABC) Scale | Not reported | Self-reported impairments, limitations, and restrictions | Not reported | 16 questions in which people with stroke rate their balance confidence during routine activities; scores range from 0–100, with higher scores indicating more confidence. Time to administer (min.): 20 |
| **Berg Balance Scale (BBS)** | Not reported | Balance | Not reported | Criterion-based assessment of static and dynamic balance; widely used in multiple settings. Time to administer (min.): 15 |
| Box and Block Test (BBT) | Not reported | UE function | Not reported | Score is the number of blocks moved in 1 min; higher scores equal better performance; normative data are available for comparison. Time to administer (min.): <5 |
| Chedoke Arm and Hand Activity Inventory (CAHAI) | Not reported | UE function | Not reported | Criterion based with functional items requiring bilateral UE movement; available in 7-, 8-, 9-, and 13-item versions. Time to administer (min.): 25 |
| Chedoke McMaster Stroke Assessment - Impairment inventory | Not reported | Sensorimotor impairment measures | Not reported | Quantifies impairments in 6 dimensions of shoulder pain, postural control, arm, hand, leg, and foot, each on a 7-point scale, with higher scores equalling less impairment. Time to administer (min.): 45 |
| Fugl-Meyer Assessment of Motor Recovery after Stroke (FMA) | Not reported | Sensorimotor impairment measures | Not reported | Quantifies sensorimotor impairment of the UE (0–66 points) and LE (0–34 points) on separate subscales; items are rated on ability to move out of abnormal synergies. Time to administer (min.): 25 |
| Functional Ambulation Categories (FAC) | Not reported | Mobility | Not reported | Classification made after observation or self-report of walking ability; 6-point scale with higher equals better walking ability; this tool allows assessment of walking ability in people who are not independent ambulators. Time to administer (min.): <5 |
| **Functional Reach Test (FRT)** | Not reported | Balance | Not reported | A single-item test that measures how far one can reach in standing; normative data are available for comparison. Time to administer (min.): <5 |
| Grip, pinch dynamometry | Not reported | Paresis/strength | Not reported | Grip and pinch dynamometers are available in most rehabilitation clinics and hospitals; normative data are available for comparison. Time to administer (min.): <5 |
| Modified Ashworth Scale (MAS) | Not reported | Tone | Not reported | Quantifies spasticity on a scale measuring resistance to passive movement from 0–4, with higher numbers indicating greater severity; can assess at all joints or only a few. Time to administer (min.): 10 |
| Motor Activity Log | Not reported | Self-reported impairments, limitations, and restrictions | Not reported | 14 or 28 questions about how the affected UE is used in daily life; scores range from 0–5, with 5 equals to similar to before the stroke. Time to administer (min.): 20 |
| Motricity Index | Not reported | Paresis/strength | Not reported | Consists of strength testing via manual muscle testing at 3 key UE segments and 3 key LE segments; yields a score from 0–100 indicating strength of each limb. Time to administer (min.): <5 for UEs; <5 for Les. |
| Muscle strength | Not reported | Paresis/strength | Not reported | Via manual muscle testing, graded on a 0–5 scale or handheld dynamometry. Time to administer (min.): <5 |
| Observational gait analysis | Not reported | Mobility | Not reported | Commonly used in many clinics to plan treatment programs; several standardized formats are available; appropriate to use in conjunction with one of the above more quantifiable measures. Time to administer (min.): 5 |
| Stroke Impact Scale: Strength, Mobility, ADL, and Hand Function subscales | Not reported | Self-reported impairments, limitations, and restrictions | Not reported | These 4 subscales measure different aspects of physical performance; people rate their perceived ability to do different items; each subscale ranges from 0–100, with higher scores indicating better abilities. Time to administer (min.): 5 per subscale |
| **Timed Up and Go Test (TUG)** | Not reported | Mobility | Not reported | Quantifies more than straight walking, including sit/stand and a turn; scored by time to complete; criterion values available for comparison. Time to administer (min.): <5 |
| **Walking speed^*^** | Not reported | Mobility | Not reported | **^*^**Generally tested on 5- or 10-m walkways. Brief and widely used; categories based on speed are: <0.4 m/s=household ambulation; 0.4–0.8 m/s=limited community ambulation >0.8 m/s=community ambulation; normative data available for comparison. Time to administer (min.): <5 |
| Wolf Motor Function Test | Not reported | UE function | Not reported | Time- and criterion-based scores on 15 items; contains some isolated joint movements and some functional tasks. Time to administer (min.): 15 |
| **6-Minute Walk Test (6mWT)** | Not reported | Mobility | Not reported | Quantifies walking endurance; normative and criterion values for community ambulation distances available. Time to administer (min.): <10 |

Abbreviations: UE: upper extremity; LE: lower extremity

| **Guideline: Guidelines for Prevention and Management of Stroke**(7) | | | | |
| --- | --- | --- | --- | --- |
| **Name and/or version of the assessment tool** | **Level of recommendation** | **Construct assessed according to the guideline** | **Time of administration recommended by the guideline** | **Resources** |
| Fugl-Meyer Assessment of Motor Recovery after Stroke (FMA) | Not reported | Functional performance | Not reported | Not reported |
| Modified Barthel Index of Activities of Daily Living (MBI) | Not reported | Basic ADL | Not reported | Not reported |

Abbreviations: ADL: activities of daily living

| **Guideline: KNGF Clinical Practice Guideline for Physical Therapy in patients with stroke**(8) | | | | |
| --- | --- | --- | --- | --- |
| **Name and/or version of the assessment tool** | **Level of recommendation** | **Construct assessed according to the guideline** | **Time of administration recommended by the guideline** | **Resources** |
| Action Research Arm Test (ARAT) | Not reported | Dexterity | (H)AR, VR, LR, RC | <https://meetinstrumentenzorg.nl/instrumenten/action-research-arm-test/> |
| Barthel Index of Activities of Daily Living (BI) | Not reported | Basic ADL | (H)AR, VR, LR, RC | <https://meetinstrumentenzorg.nl/instrumenten/barthel-index/> |
| **Berg Balance Scale (BBS)** | Not reported | Sitting and standing balance | (H)AR, VR, LR, RC | <https://meetinstrumentenzorg.nl/instrumenten/berg-balance-scale/> |
| Caregiver Strain Index (CSI) | Not reported | Caregiver strain | VR, LR, RC | Not reported |
| Cumulative Illness Rating Scale (CIRS) | Not reported | Multimorbidity | (H)AR, VR, LR, RC | Not reported |
| Erasmus Nottingham Sensory Assessment (EmNSA) | Not reported | Somatosensory impairments | (H)AR, VR, LR, RC | <https://meetinstrumentenzorg.nl/instrumenten/erasmus-mc-modificatie-van-de-revised-nottigham-sensory-assessment/> |
| Falls Efficacy Scale (FES) | Not reported | Self-efficacy in maintaining balance | (H)AR, VR, LR, RC | <https://meetinstrumentenzorg.nl/instrumenten/falls-efficacy-scale-international-7-16-items/> |
| Fatigue Severity Scale (FSS) | Not reported | Fatigue | LR, RC |  |
| Fugl-Meyer Assessment of Motor Recovery after Stroke (FMA) (LE) | Not reported | Walking and walking-related functions and activities; Muscle strength | (H)AR, VR, LR, RC | <https://meetinstrumentenzorg.nl/instrumenten/fugl-meyer-assessment/> |
| Fugl-Meyer Assessment of Motor Recovery after Stroke (FMA) (UE**)** | Not reported | Dexterity and related functions and activities - Selective movements | (H)AR, VR, LR, RC | <https://meetinstrumentenzorg.nl/instrumenten/fugl-meyer-assessment/> |
| Functional Ambulation Categories (FAC) | Not reported | Walking ability | (H)AR, VR, LR, RC | <https://meetinstrumentenzorg.nl/instrumenten/functional-ambulation-classification/> |
| Frenchay Arm Test (FAT) | Not reported | Dexterity | (H)AR, VR, LR, RC | <https://meetinstrumentenzorg.nl/instrumenten/frenchay-arm-test/> |
| Hospital Anxiety and Depression Scale (HADS) | Not reported | Anxiety and depression | VR, LR, RC | Not reported |
| Modified Ashworth Scale (MAS) | Not reported | Resistance to passive movements | (H)AR, VR, LR, RC | Not reported |
| Montreal Cognitive Assessment (MoCA) | Not reported | Cognitive functions | (H)AR, VR, LR, RC | Not reported |
| Motricity Index (UE/ LE) | Not reported | Muscle strength | (H)AR, VR, LR, RC | Not reported |
| National Institutes of Health Stroke Scale (NIHSS) | Not reported | Neurological impairments | (H)AR, VR | https://meetinstrumentenzorg.nl/instrumenten/national-institutes-of-health-stroke-scale/ |
| Neutral-zero method (NZM) (goniometer) | Not reported | Range of motion | (H)AR, VR, LR, RC | Not reported |
| Nine Hole Peg test | Not reported | Dexterity | (H)AR, VR, LR, RC | <https://meetinstrumentenzorg.nl/instrumenten/nine-hole-peg-test/> |
| Nottingham extended ADL index | Not reported | Extended ADL | (H)AR, VR, LR, RC | <https://meetinstrumentenzorg.nl/instrumenten/nottingham-extended-activities-of-daily-living-index/> |
| Numeric Pain Rating Scale (NPRS) | Not reported | Pain experienced | (H)AR, VR, LR, RC | Not reported |
| O-Letter Cancellation Test (O-LCT) | Not reported | Neglect | (H)AR, VR, LR, RC | Not reported |
| Stroke-Specific Quality of Life scale (SSQoL) | Not reported | Quality of life | RC | Not reported |
| **Timed Up and Go Test (TUG)** | Not reported | Walking ability | (H)AR, VR, LR, RC | <https://meetinstrumentenzorg.nl/instrumenten/timed-up-go-test-2/> |
| **Trunk Control Test (TCT)** | Not reported | Trunk activity | (H)AR, VR, LR, RC | <https://meetinstrumentenzorg.nl/instrumenten/trunk-control-test/> |
| **Trunk Impairment Scale (TIS)** | Not reported | Sitting balance | (H)AR, VR, LR, RC | <https://meetinstrumentenzorg.nl/instrumenten/trunk-impairment-scale/> |
| **6-Minute Walk Test (6MWT)** | Not reported | Walking distance, functional endurance | (H)AR, VR, LR, RC | <https://meetinstrumentenzorg.nl/instrumenten/6-minute-walk-test-zes-minuten-wandeltest/> |
| **10-Meter Walk Test (10mWT)** | Not reported | Walking speed (at comfortable and maximum walking speed) | (H)AR, VR, LR, RC | <https://meetinstrumentenzorg.nl/instrumenten/10-meter-walk-test-tien-meter-looptest/> |

Abbreviations: (H)AR: Hyperacute/acute (rehabilitation) phase, lasting 0 to 24 hours; ER: Early rehabilitation phase, lasting from 24 hours to 3 months; LR: Late rehabilitation phase, lasting 3 to 6 months; RC: Rehabilitation in the chronic phase, lasting longer than 6 months; ADL: activities of daily living

| **Guideline: Philippine Academy of Rehabilitation Medicine (PARM): Clinical Practice Guideline on Stroke Rehabilitation (Updated: 2017)**(9) | | | | |
| --- | --- | --- | --- | --- |
| **Name and/or version of the assessment tool** | **Level of recommendation** | **Construct assessed according to the guideline** | **Time of administration recommended by the guideline** | **Resources** |
| Aphasia Depression Rating Scale (ADRS) | Not reported | Depression | Not reported | Not reported |
| Beck Depression Inventory (BDI) | Not reported | Depression | Not reported | Not reported |
| **Berg Balance Scale (BBS)** | Not reported | Level of assistance for mobility and self-care. | Not reported | Not reported |
| Canadian Occupational Performance Measure | Not reported | Maintained functional independence and optimal participation. | Not reported | Not reported |
| Center for Epidemiological Studies Depression Scale (CES-D) | Not reported | Depression | Not reported | Not reported |
| Functional Independence Measure (FIM) mobility items | Not reported | Level of assistance for mobility and self-care. | Not reported | Not reported |
| Functional Ambulation Classification (FAC) | Not reported | Assistance needed during daily activities | Not reported | Not reported |
| Frenchay Activities Index | Not reported | Instrumental ADL, leisure, and participation | Not reported | Not reported |
| Geriatric Depression Scale (GDS) | Not reported | Depression | Not reported | Not reported |
| Hospital Anxiety and Depression Scale (HADS) | Not reported | Mood alterations | Not reported | Not reported |
| Montreal Cognitive Assessment | Not reported | Cognitive impairment | Not reported | Provides administration and scoring instructions |
| Naturalistic Action Test | Not reported | Difficulties executing tasks | Not reported | For more information regarding the test, visit the Moss Rehabilitation Research Institute website at http://mrri.org/naturalistic-action-test/ |
| Post-Stroke Depression Predict Scale (DePres) | Not reported | Depression | Not reported | Not reported |
| **10-Meter Walk Test (10mWT)** | Not reported | Gait velocity | Not reported | Not reported |

Abbreviations: ADL: activities of daily living

| **Guideline: Rehabilitation von sensomotorischen Störungen, S2k-Leitlinie**(10) | | | | |
| --- | --- | --- | --- | --- |
| **Name and/or version of the assessment tool** | **Level of recommendation** | **Construct assessed according to the guideline** | **Time of administration recommended by the guideline** | **Resources^*^** |
| Action Research Arm Test (ARAT) | Not reported | Ability to grasp and manipulate | NR |  |
| Ashworth Scale | Not reported | Spasticity | Not reported |  |
| Assessment of Motor and Process Skills (AMPS) | Not reported | ADL | Not reported |  |
| Barthel Index of Activities of Daily Living (BI) | Not reported | ADL | Not reported |  |
| **Berg Balance Scale (BBS)** | Not reported | Postural control in transitions from standing up to walking | Not reported |  |
| BMRC test | Not reported | Paresis/Strength | Not reported |  |
| **Bohannon-Balance-Test** | Not reported | Postural control while standing | Not reported |  |
| Box and Block Test (BBT) | Not reported | Ability to grasp and manipulate | Not reported |  |
| Canadian Occupational Performance Measure (COPM) | Not reported | Disability and quality of life | Not reported |  |
| **Dynamic Gait Index (DGI)** | Not reported | Locomotion | Not reported |  |
| Dynamometry | Not reported | Paresis/Strength | Not reported |  |
| EQ-5D (EuroQol: health-related quality of life) | Not reported | Disability and quality of life | Not reported |  |
| Frenchay Arm Test | Not reported | Ability to grasp and manipulate | Not reported |  |
| Fugl-Meyer Assessment of Motor Recovery after Stroke (FMA) | Not reported | Motor function | Not reported |  |
| Fugl-Meyer Assessment of Motor Recovery after Stroke (FMA) - Arm section | Not reported | Ability to grasp and manipulate | Not reported |  |
| Functional Ambulation Categories (FAC) | Not reported | Locomotion | Not reported |  |
| Functional Independence Measure (FIM) | Not reported | ADL | Not reported |  |
| **Functional Reach Test (FRT)** | Not reported | Postural control/ risk of falling | Not reported |  |
| Frühreha-Barthel-Index (FBI) | Not reported | ADL | Not reported |  |
| Jebsen-Taylor-Hand function test | Not reported | Ability to grasp and manipulate | Not reported |  |
| Modified Ashworth Scale (MAS) | Not reported | Spasticity | Not reported |  |
| Motor Activity Log (MAL) | Not reported | Ability to grasp and manipulate | Not reported |  |
| **Motor Assessment Scale (MAS)** | Not reported | Motor function | Not reported |  |
| **Motor Club Assessment (MCA)** | Not reported | Motor function | Not reported |  |
| Motricity Index | Not reported | Motor function | Not reported |  |
| National Institutes of Health Stroke Scale | Not reported | General function | Not reported |  |
| Nottingham Assessment of Somatosensation (NAS) | Not reported | Sensibility | Not reported |  |
| Nottingham Extended ADL (NEADL) | Not reported | ADL | Not reported |  |
| Passive range of motion (PROM) | Not reported | Muscle length | Not reported |  |
| Pendel Test | Not reported | Spasticity | Not reported |  |
| Rivermead Assessment of Somatosensory Performance (RASP) | Not reported | Sensibility | Not reported |  |
| Rivermead Mobility Index | Not reported | Locomotion | Not reported |  |
| SF-36 | Not reported | Disability and quality of life | Not reported |  |
| Stroke Impact Scale (SIS) | Not reported | General function; Disability and quality of life | Not reported |  |
| Stroke Specific Quality of Life Scale (SS-QOL) | Not reported | Disability and quality of life | Not reported |  |
| Tardieu Scale | Not reported | Spasticity | Not reported |  |
| **Timed Up and Go Test (TUG)** | Not reported | locomotion | Not reported |  |
| **Trunk Control Test (TCT)** | Not reported | Postural control (only trunk control) | Not reported |  |
| Wolf Motor Function Test | Not reported | Ability to grasp and manipulate | Not reported |  |
| **6-Minute Walk Test (6mWT)** | Not reported | Locomotion | Not reported |  |
| **10-Meter Walk Test (10mWT)** | Not reported | Locomotion | Not reported |  |

^*^Provides reference to this book obtain detailed descriptions of the tools: Masur H et al., Hrsg. Skalen und Scores in der Neurologie, 2. Aufl. Stuttgart: Thieme, 2000.(11)

Abbreviations: ADL: activities of daily living

| **Guideline: South African-contextualised stroke rehabilitation guideline (SA-CSRG)**(12) | | | | |
| --- | --- | --- | --- | --- |
| **Name and/or version of the assessment tool** | **Level of recommendation** | **Construct assessed according to the guideline** | **Time of administration recommended by the guideline** | **Resources** |
| Action Research Arm Test (ARAT) | Not reported | UE function | Not reported | Criteria based with 19 items; scores are from 0–57, with normal=57; allows observation of multiple grasps, grips, and pinches. Time to administer (min.): 10  Agreed minimal important difference: 12 and 17 points for the affected dominant and non-dominant sides respectively |
| Activities-specific Balance Confidence (ABC) Scale | Not reported | Self-reported impairments, limitations, and restrictions | Not reported | 16 questions in which people with stroke rate their balance confidence during routine activities; scores range from 0–100, with higher scores indicating more confidence. Time to administer (min.): 20 |
| Barthel Index of Activities of Daily Living (BI) | Not reported | Not reported | Not reported | Agreed minimal important difference: 1.85 (SE: 1.45) |
| **Berg Balance Scale (BBS)** | Not reported | Balance | Not reported | Criterion-based assessment of static and dynamic balance; widely used in multiple settings. Time to administer (min.): 15 |
| Box and Block Test (BBT) | Not reported | UE function | Not reported | Score is the number of blocks moved in 1 min; higher scores equal better performance; normative data are available for comparison. Time to administer (min.): <5 |
| Chedoke Arm and Hand Activity Inventory (CAHAI) | Not reported | UE function | Not reported | Criterion based with functional items requiring bilateral UE movement; available in 7-, 8-, 9-, and 13-item versions. Time to administer (min.): 25 |
| Chedoke McMaster Stroke Assessment - Impairment inventory | Not reported | Sensorimotor impairment measures | Not reported | Quantifies impairments in 6 dimensions of shoulder pain, postural control, arm, hand, leg, and foot, each on a 7-point scale, with higher scores equalling less impairment. Time to administer (min.): 45 |
| Fugl-Meyer Assessment of Motor Recovery after Stroke (FMA) | Not reported | Sensorimotor impairment measures | Not reported | Quantifies sensorimotor impairment of the UE (0–66 points) and LE (0–34 points) on separate subscales; items are rated on ability to move out of abnormal synergies. Time to administer (min.): 25  Agreed minimal important difference: difference by 10% of the total score |
| Functional Ambulation Categories (FAC) | Not reported | Mobility | Not reported | Classification made after observation or self-report of walking ability; 6-point scale with higher equals better walking ability; this tool allows assessment of walking ability in people who are not independent ambulators. Time to administer (min.): <5 |
| Functional Independence Measure (FIM) | Not reported | Not reported | Not reported | Agreed minimal important difference: 22 points for the total FIM, 17 points (on the 105 point scale – 16%) for the motor FIM and 3 points for the cognitive FIM |
| **Functional Reach Test (FRT)** | Not reported | Balance | Not reported | A single-item test that measures how far one can reach in standing; normative data are available for comparison. Time to administer (min.): <5 |
| Grip, pinch dynamometry | Not reported | Paresis/strength | Not reported | Grip and pinch dynamometers are available in most rehabilitation clinics and hospitals; normative data are available for comparison. Time to administer (min.): <5 |
| Modified Ashworth Scale (MAS) | Not reported | Tone | Not reported | Quantifies spasticity on a scale measuring resistance to passive movement from 0–4, with higher numbers indicating greater severity; can assess at all joints or only a few. Time to administer (min.): 10 |
| Motor Activity Log | Not reported | Self-reported impairments, limitations, and restrictions | Not reported | 14 or 28 questions about how the affected UE is used in daily life; scores range from 0–5, with 5 equals to similar to before the stroke. Time to administer (min.): 20  Agreed minimal important difference: at least 1.0 and 1.1 points (17-18% of the scale) for the affected dominant and non-dominant sides respectively |
| Motricity Index | Not reported | Paresis/strength | Not reported | Consists of strength testing via manual muscle testing at 3 key UE segments and 3 key LE segments; yields a score from 0–100 indicating strength of each limb. Time to administer (min.): <5 for UEs; <5 for Les. |
| Muscle strength | Not reported | Paresis/strength | Not reported | Via manual muscle testing, graded on a 0–5 scale or handheld dynamometry. Time to administer (min.): <5 |
| Observational gait analysis | Not reported | Mobility | Not reported | Commonly used in many clinics to plan treatment programs; several standardized formats are available; appropriate to use in conjunction with one of the above more quantifiable measures. Time to administer (min.): 5 |
| Stroke Impact Scale: Strength, Mobility, ADL, and Hand Function subscales | Not reported | Self-reported impairments, limitations, and restrictions | Not reported | These 4 subscales measure different aspects of physical performance; people rate their perceived ability to do different items; each subscale ranges from 0–100, with higher scores indicating better abilities. Time to administer (min.): 5 per subscale |
| **Timed Up and Go Test (TUG)** | Not reported | Mobility | Not reported | Quantifies more than straight walking, including sit/stand and a turn; scored by time to complete; criterion values available for comparison. Time to administer (min.): <5  Agreed minimal important difference: 10 seconds |
| **Walking speed^*^** | Not reported | Mobility | Not reported | **^*^**Generally tested on 5- or 10-m walkways. Brief and widely used; categories based on speed are: <0.4 m/s=household ambulation; 0.4–0.8 m/s=limited community ambulation >0.8 m/s=community ambulation; normative data available for comparison. Time to administer (min.): <5 |
| Wolf Motor Function Test | Not reported | UE function | Not reported | Time- and criterion-based scores on 15 items; contains some isolated joint movements and some functional tasks. Time to administer (min.): 15  Agreed minimal important difference: an improvement of 19 seconds on the affected dominant side (16% of the 120 second limit) |
| **6-Minute Walk Test (6MWT)** | Not reported | Mobility | Not reported | Quantifies walking endurance; normative and criterion values for community ambulation distances available. Time to administer (min.): <10  Agreed minimal important difference: 28m |

Abbreviations: UE: upper extremity; LE: lower extremity

**REFERENCES**

1. Haute Autorité de Santé (HAS). Accident vasculaire cérébral. Pertinence des parcours de rééducation/réadaptation après la phase initiale de l’AVC. Note de problématique. Saint Denis La Plaine HAS [Internet]. 2019; Available from: https://www.has-sante.fr/jcms/c_2972905/fr/accident-vasculaire-cerebral-pertinence-des-parcours-de-reeducation/readaptation-apres-la-phase-initiale-de-l-avc

2. Moore JL, Potter K, Blankshain K, Kaplan SL, O’Dwyer LC, Sullivan JE. A core set of outcome measures for adults with neurologic conditions undergoing rehabilitation. Vol. 42, Journal of Neurologic Physical Therapy. 2018.

3. Teasell R, Salbach NM, Foley N, Mountain A, Cameron JI, Jong A de, et al. Canadian Stroke Best Practice Recommendations: Rehabilitation, Recovery, and Community Participation following Stroke. Part One: Rehabilitation and Recovery Following Stroke; 6th Edition Update 2019. Int J Stroke. 2020;15(7).

4. Gesellschaft D. DEGAM Leitlinie-S3: Schlaganfall. (053). Available from: https://www.degam.de/files/Inhalte/Leitlinien-Inhalte/Dokumente/DEGAM-S3-Leitlinien/053-011_Schlaganfall/053-011l_LL_Schlaganfall.pdf

5. Asociación Colombiana de Fisioterapia (ASCOFI) - Asociación Colombiana de Facultades de Fisioterapia (ASCOFAFI). Guía de práctica clínica fisioterapéutica para la evaluación y tratamiento de pacientes con enfermedades cerebrovasculares en los primeros seis meses de la enfermedad. 2021;1–21.

6. Winstein CJ, Stein J, Arena R, Bates B, Cherney LR, Cramer SC, et al. Guidelines for Adult Stroke Rehabilitation and Recovery: A Guideline for Healthcare Professionals from the American Heart Association/American Stroke Association. Vol. 47, Stroke. 2016.

7. Directorate General of Health Services Ministry of Health and Family Welfare Government of India. Guidelines for Prevention and Management of Stroke. 2019 [cited 2021 Jun 13]; Available from: https://main.mohfw.gov.in/sites/default/files/Guidelines for Prevention and Managment of Stroke.pdf

8. Royal Dutch Society for Physical Therapy. KNGF Clinical Practice Guideline for Physical Therapy in patients with stroke. Pract Guidel. 2014;04(1).

9. Philippine Academy of Rehabilitation Medicine (PARM). Philippine Academy of Rehabilitation Medicine (PARM): Clinical Practice Guidelines on Stroke Rehabilitation. 2019;1–278. Available from: https://parm.org.ph/pdf/strokerehab.pdf

10. Nelles G. Rehabilitation von sensomotorischen Störungen. Leitlinien für Diagnostik und Ther der Neurol. 2018;1–45.

11. Masur H et al. Skalen und Scores in der Neurologie. Thieme, Stuttgart;

12. SA-CSRG. South African- Contextualised Stroke Rehabilitation Guideline (Sa-Csrg). Med Sci. 2019;15(January).
